# Outer Membrane Vesicles Displaying a Heterologous PcrV-HitA Fusion Antigen Promote Protection against Pulmonary Pseudomonas aeruginosa Infection

**DOI:** 10.1128/mSphere.00699-21

**Published:** 2021-10-06

**Authors:** Peng Li, Xiuran Wang, Xiangwan Sun, Ziqiang Guan, Wei Sun

**Affiliations:** a Department of Immunology and Microbial Disease, Albany Medical College, Albany, New York, USA; b Department of Biochemistry, Duke University Medical Center, Durham, North Carolina, USA; U.S. Food and Drug Administration

**Keywords:** *P. aeruginosa*, outer membrane vesicles, heterologous antigen, vaccine, protective immunity

## Abstract

Along with surging threats and antibiotic resistance of Pseudomonas aeruginosa in health care settings, it is imperative to develop effective vaccines against P. aeruginosa infection. In this study, we used an Asd (aspartate-semialdehyde dehydrogenase)-based balanced-lethal host-vector system of a recombinant Yersinia pseudotuberculosis mutant to produce self-adjuvanting outer membrane vesicles (OMVs). The OMVs were used as a carrier to deliver the heterologous PcrV-HitA_T_ (PH) fusion antigen of P. aeruginosa for vaccine evaluation. Intramuscular vaccination with OMVs carrying the PH antigen (referred to rOMV-PH) afforded 73% protection against intranasal challenge with 5 × 10^6^ (25 50% lethal doses) of the cytotoxic PA103 strain and complete protection against a noncytotoxic PAO1 strain. In contrast, vaccination with the PH-deficient OMVs or PH antigen alone failed to offer effective protection against the same challenge. Immune analysis showed that the rOMV-PH vaccination induced potent humoral and Th1/Th17 responses compared to the PH vaccination. The rOMV-PH vaccination rapidly cleared P. aeruginosa burdens with coordinated production of proinflammatory cytokines in mice. Moreover, antigen-specific CD4^+^ and CD8^+^ T cells and their producing cytokines (tumor necrosis factor alpha and interleukin-17A), rather than antibodies, were essential for protection against pneumonic P. aeruginosa infection. Our studies demonstrated that the recombinant Y. pseudotuberculosis OMVs delivering heterologous P. aeruginosa antigens could be a new promising vaccine candidate for preventing the spread of drug-resistant P. aeruginosa.

**IMPORTANCE** Hospital- and community-acquired infections with Pseudomonas aeruginosa cause a high rate of morbidity and mortality in patients who have underlying medical conditions. The spread of multidrug-resistant P. aeruginosa strains is becoming a great challenge for treatment using antibiotics. Thus, a vaccine as one of the alternative strategies is urgently required to prevent P. aeruginosa infection.

## INTRODUCTION

Pseudomonas aeruginosa, a Gram-negative opportunistic bacterial pathogen (1), is one of the leading pathogens responsible for life-threatening pneumonia and systemic infection (2), especially among immunocompromised subjects with underlying diseases such as cancer, AIDS (3), or cystic fibrosis (CF) (4). Additionally, P. aeruginosa is especially problematic to long-term hospitalized patients in intensive care units and burn victims (5). Ventilator-associated pneumonia caused by P. aeruginosa accounts for 11% of total cases (6, 7) and contributes to mortality rates as high as 13.5% (8). P. aeruginosa infection in burn patients can quickly develop into systemic infection, with mortality rates ranging between 38% and 70% (9). Due to a complex gene regulation network, P. aeruginosa can rapidly develop resistance to a variety of antibiotics, such as aminoglycosides, quinolones, and β-lactams, through intrinsic acquired and adaptive manners (10), leading to extreme challenges for counteracting P. aeruginosa infection (11, 12). Since resistance rates of P. aeruginosa are increasing in many parts of the world, multiple-drug-resistant P. aeruginosa is listed as one of the most serious threats in CDC reports (13).

Due to a paucity of antipseudomonal antibiotics, vaccination against P. aeruginosa is considered one of the most effective alternatives in eliminating and reducing the need for antibiotic agents as well as in combating the spread of drug-resistant P. aeruginosa (6). In the past few decades, vigorous studies have been conducted in pursuit of an effective P. aeruginosa vaccine; however, no licensed vaccines are currently available for humans (14). Bacterial outer membrane vesicles (OMVs) naturally containing immunomodulating components can stimulate host innate and adaptive immunity (15). As nanoscale particles, OMVs have intrinsic advantages in promoting uptake by antigen-presenting cells (APCs), resulting in increased immunogenicity (16). Recently, a licensed vaccine containing OMVs from Neisseria meningitidis has been proven safe and has great protection against N. meningitidis serogroup b in humans (17), which incites wide enthusiasm for developing OMV vaccines against different pathogens (18, 19). A growing body of evidence has shown that mice immunized with OMVs containing specific heterologous antigens generate protective responses against infection of pathogens that possess the heterologous antigens (20, 21). Recently, we used a self-adjuvanting bacterial OMV derived from recombinant Yersinia pseudotuberculosis (strain Yptb) to deliver the Y. pestis LcrV antigen as a vaccine. The OMV vaccination offered excellent protection for mice against both pneumonic and bubonic plague (unpublished data). Thus, we hypothesize that this platform is viable in delivering P. aeruginosa antigens for preventing P. aeruginosa infection.

PcrV is a conserved protein with ∼98% identity among different serotypes of PA isolates and a promising antigen candidate (6). In P. aeruginosa, the PcrV protein forms a ring structure at the tip of the needle of the type three secretion system (T3SS) and is essential for translocation of the effectors (22) and bacterial pathogenicity (23). Immunization with recombinant PcrV or passive transfer of anti-PcrV antibodies offered significant protection against lethal P. aeruginosa infections (24, 25). In addition, iron is an indispensable nutrient for the replication of almost all bacteria (26). Multiple iron acquisition systems are used by P. aeruginosa to obtain iron from mammalian hosts during infection and play an important role in bacterial virulence (27). P. aeruginosa *hitA* (PA4687) and *hitB* (PA4688), encoding ferric iron-binding periplasmic proteins, are involved in iron transport and are associated with bacterial virulence (28). A study showed that immunization with HitA afforded protection against P. aeruginosa infection in mice (29). Protein alignment indicates that HitA is a highly conserved protein with ∼100% identity among all sequenced P. aeruginosa strains. Therefore, immunization with Yptb OMVs delivering heterologous PcrV and HitA antigens as a bivalent vaccine may offer significant protective immunity against pulmonary infection of P. aeruginosa.

In this study, we used an Asd (aspartate-semialdehyde dehydrogenase)-based balanced-lethal recombinant Yptb system tailored with an Asd^+^ plasmid to oversynthesize the heterologous truncated PcrV-HitA_T_ fusion antigen (referred to as PH) and produce large amounts of OMVs encasing the PH antigen. Intramuscular (i.m.) immunization with rOMV-PH offered significant protection against lethal intranasal (i.n.) challenge with the PA103 or PAO1 strain and stimulated robust antigen-specific B- and T-cell responses.

## RESULTS

### OMVs displaying the heterologous PcrV-HitA_T_ fusion antigen of P. aeruginosa.

**FIG 1:**
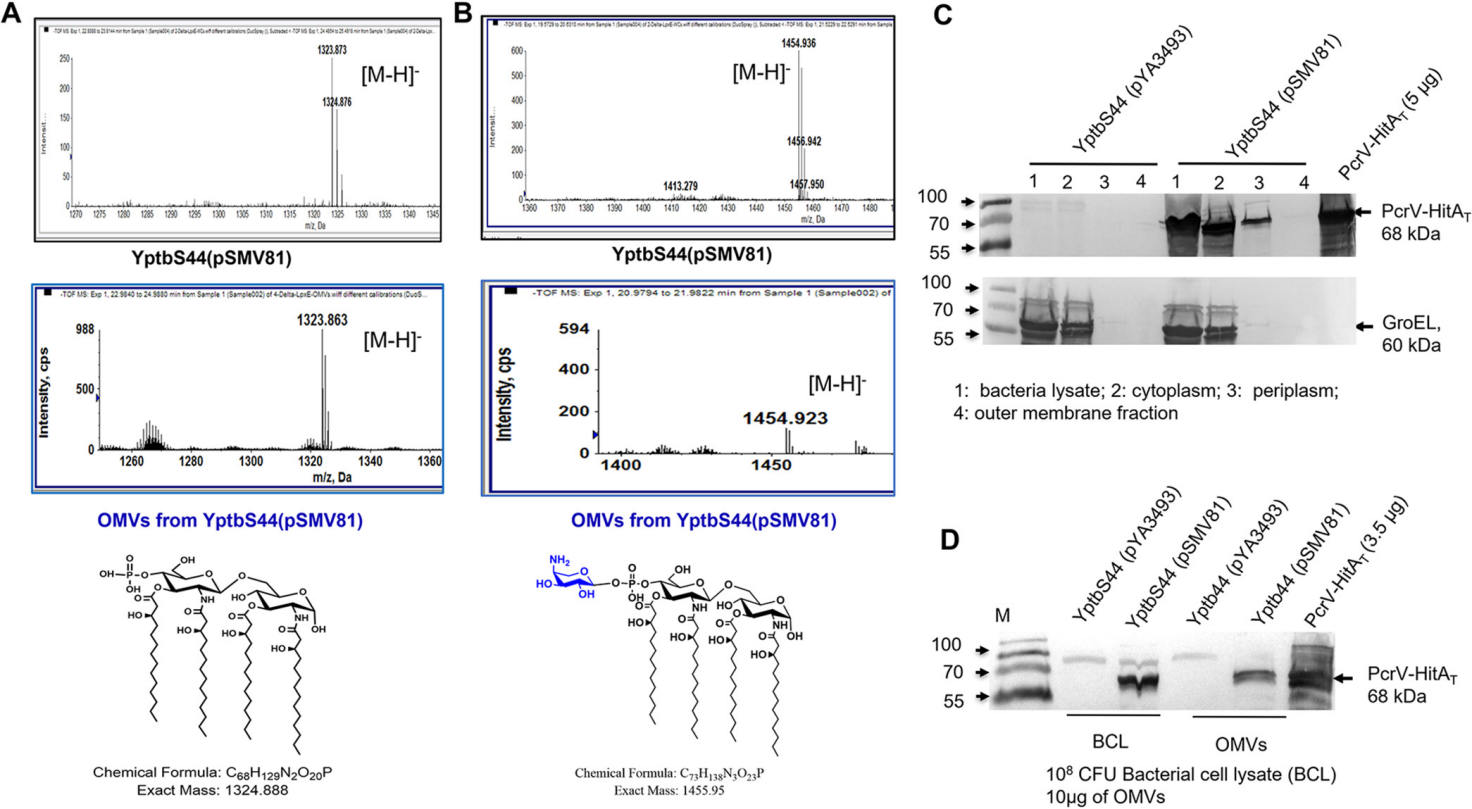
Lipid A and the P. aeruginosa fusion antigen analysis of recombinant Y. pseudotuberculosis and its outer membrane vesicles (rOMVs). (A and B) Mass spectrometry analysis of lipid A species in the YptbS44(pSMV81) strain and its OMVs. 1-Dephosphorylated tetra-acylated lipid A species are present in the YptbS44(pSMV81) strain and its OMVs. (C) The presence of PcrV-HitA_T_ (PH) fusion antigen (PH) was determined in different cellular fractions (whole bacterial lysate, cytoplasmic fraction, periplasmic fraction, and outer membrane fraction) of the YptbS44(pSMV81) strain. (D) Bacterial cell lysate (BCL) or OMVs isolated from YptbS44 harboring empty plasmid pYA3493 or pSMV81 were examined for the presence of the PH fusion protein by mouse anti-PcrV antibody (1:5,000) using immunoblotting. Each well was loaded with BCL prepared from 10^8^ CFU of bacteria or 10 μg rOMVs. M, molecular size marker.

A hypervesiculating Y. pseudotuberculosis mutant strain, YptbS44 (Table 1; see also Table S1 and Text S1 in the supplemental material), was used. YptbS44 was tailored with an Asd^+^ plasmid, pSMV81 (Table 1, Fig. S1A), in which the *pcrV-hitA_T_* fusion DNA fragment was ligated with the N-terminal β-lactamase signal sequence (*bla ss*) to facilitate secretion of PcrV-HitA_T_ (PH) into bacterial periplasm by the type II secretion system (30). Lipid A species of the YptbS44(pSMV81) strain and its OMVs (Fig. S1B) were analyzed using mass spectrometry. Results showed that lipid A profiles in bacteria and OMVs were similar and contained both monophosphoryl lipid A (MPLA) (Fig. 1A and B) and hexa-acylated lipid A (endotoxin) (Fig. S1C). It appeared that the peak of MPLA with l-Ara4N modification in OMVs was lower than that in the strain (Fig. 1B). Further, we compared the secreted embryonic alkaline phosphatase (SEAP) activity of HEK-Blue mTLR4 cells cultured with different OMVs. The TLR4 stimulatory activities of OMVs from YptbS44 harboring pSMV81 or an empty plasmid were significantly lower than OMVs from the Salmonella enterica serovar Typhimurium Δ*msbB* mutant (a positive control) but still significantly higher than the purified PH fusion protein and phosphate-buffered saline (PBS) controls (Fig. S1D).

**TABLE 1:** Strains and plasmids used in this study

Strain or plasmid	Genotype or relevant characteristics	Source or reference
Strains		
E. coli χ6212	*F^−^ λ^−^ ϕ80* Δ(*lacZYA-argF*) *endA1 recA1 hsdR17 deoR thi-1 glnV44 gyrA96 relA1* Δ*asdA4*	70
P. aeruginosa		
PA103	Wild-type strain, serogroup O11	Received from Joanna B. Goldberg
PAO1	Wild-type strain, serogroup O5	Received from Shouguang Jin
Yersinia pseudotuberculosis YptbS44	Δ*asd* Δ*tolR* Δ*hmsHFRS* Δ*lacI*::P_lpp_ *lpxE* Δ*lacZ*::*caf1R-caf1M-caf1A-caf1* pYV-	Lab collection
Plasmids		
pYA3342	Asd^+^ vector, P_trc_, pBR ori	70
pYA3493	Asd^+^ vector with β-lactamase N-terminal signal sequence, P_trc_, pBR ori	70
pSMV67	*pcrV*-6×His fragment was cloned into sites of NcoI and HindIII in the pYA3342	This study
pSMV81	*pcrV-hitA_T_* DNA fragment was cloned into sites of EcoRI and HindIII in the pYA3494	This study
pSMV82	*pcrV-hitA_T_*-6×His fragment was cloned into sites of NcoI and HindIII in the pYA3342	This study

10.1128/mSphere.00699-21.1 FIG S1 Lipid A and the PH fusion antigen analysis of recombinant Y. pseudotuberculosis and its outer membrane vesicles (rOMVs). (A) Map of plasmid pSMV81 that contains the *pcrV*-*hitA_T_* fusion gene encoding the PcrV-HitA_T_ (PH) fusion antigen. (B) Image of rOMVs using transmission electron microscopy (TEM). The rOMVs were purified from culture supernatants of recombinant Y. pseudotuberculosis strain YptbS44 harboring pSMV81 plasmid. Bars, 500 nm. (C) Mass spectrometric analysis of lipid A species in the YptbS44(pSMV81) strain and its OMVs. Hexa-acylated lipid A species (endotoxin) are present in the YptbS44(pSMV81) strain and its OMVs. (D) TLR4 activation of OMVs *in vitro*. Comparison of the secreted embryonic alkaline phosphatase (SEAP) activities in HEK-Blue cells with or without murine TLR4. HEK-Blue mTLR4 (InvivoGen) cells were cocultured with 10 μg/ml OMVs from YptbS44(pSMV81) designated rOMV-PH and 10 μg/ml of OMVs from YptbS44(pYA3493) designated rOMV-N for 8 h, respectively. The OMVs from the Salmonella Typhimurium Δ*msbB* strain was used as a positive control and 10 μg/ml of purified PcrV-HitA_T_ (PH) protein and PBS as negative controls. (E) Analysis of the purified PH fusion protein and the residual endotoxin. The purified PH protein was determined by Coomassie blue protein SDS-PAGE gel staining. The residual endotoxin in the purified PH protein was measured by the Pierce chromogenic endotoxin quant kit (Thermo Fisher Scientific). The statistical significance among the groups was analyzed by two-way multivariant ANOVA with a Tukey *post hoc* test. ns, no significance; *, *P* < 0.05; **, *P* < 0.01; ***, *P* < 0.001; ****, *P* < 0.0001. Download FIG S1, PDF file, 0.3 MB. Copyright © 2021 Li et al. 2021 Li et al. https://creativecommons.org/licenses/by/4.0/ This content is distributed under the terms of the Creative Commons Attribution 4.0 International license.

10.1128/mSphere.00699-21.5 TABLE S1 Primers for amplifying the pcrV-hitAT fusion gene fragment. Download Table S1, DOC file, 0.1 MB. Copyright © 2021 Li et al. 2021 Li et al. https://creativecommons.org/licenses/by/4.0/ This content is distributed under the terms of the Creative Commons Attribution 4.0 International license.

10.1128/mSphere.00699-21.6 TEXT S1 Supplemental Materials and Methods and references. Download Text S1, DOC file, 0.1 MB. Copyright © 2021 Li et al. 2021 Li et al. https://creativecommons.org/licenses/by/4.0/ This content is distributed under the terms of the Creative Commons Attribution 4.0 International license.

Additionally, YptbS44(pSMV81) was found to synthesize large amounts of PH (molecular mass, 68 kDa) in bacterial cell lysates (BCL), and no PH was present in YptbS44 harboring an empty plasmid pYA3493 (Fig. 1C). To examine the subcellular location of PH, different fractions of YptbS44 harboring pSMV81, such as the cytoplasm, periplasm, and outer membrane were prepared. The PH fusion antigen was detected in the bacterial cytoplasmic and periplasmic fractions but not in the outer membrane fraction (Fig. 1C), which suggests that the PH fusion antigen is located in the lumen of OMVs instead of on the surface of OMVs. OMVs from YptbS44(pSMV81) carried considerable amounts of PH fusion antigen (Fig. 1C and D). The yield of OMVs was ∼1.8 mg from 1 liter of YptbS44(pSMV81) culture and 1 μg of OMVs contained around 0.1 μg of PH titrated by the indicated amounts of purified PH fusion protein (Fig. S1E).

### Immunization with OMVs enclosing PH antigen afforded significant protection against P. aeruginosa infection.

**FIG 2:**
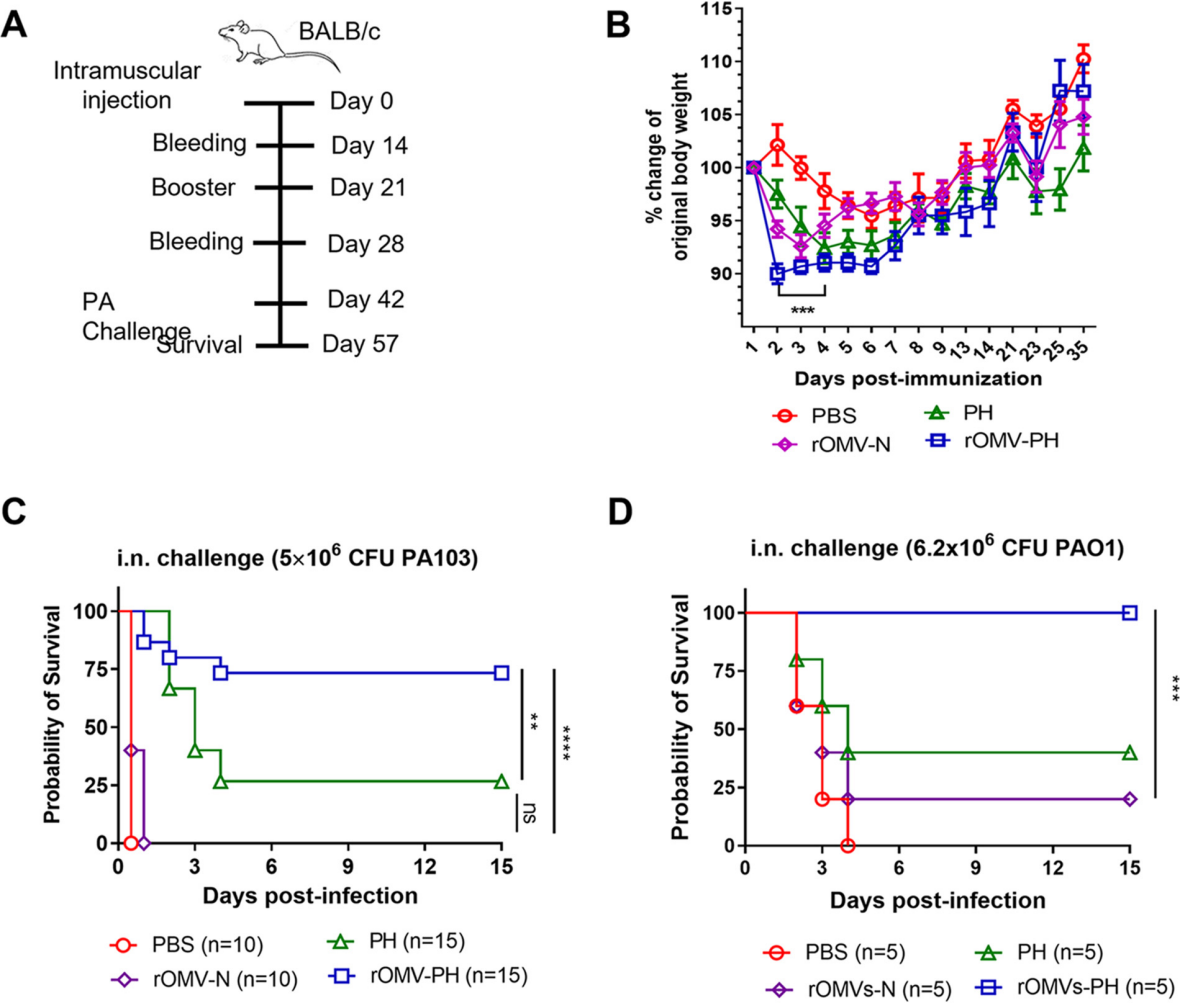
Immunization and protective efficacy of mice challenged by P. aeruginosa PA103. Groups of BALB/c mice (*n* = 10 to 15, nearly equal numbers of males and females) were intramuscularly immunized with 100 μl PBS containing 50 μg of rOMVs, 10 μg of PH-alhydrogel, or alhydrogel alone as negative controls and then boosted one time 21 days after prime immunization. Fifty micrograms of OMVs from YptbS44(pSMV81) contain ∼5 μg of PH fusion antigen. (A) Scheme of immunization regimen. (B) Rates of mouse body-weight change. (C) On 42 days after initial immunization, mice were intranasally challenged with 5 × 10^6^ CFU of the PA103 strain (25 LD_50_). The experiments were performed twice, and data were combined for analysis. (D) At 42 days after initial immunization, mice were intranasally challenged with 6.2 × 10^6^ CFU of the PAO1 strain. Statistical significance was analyzed by log-rank (Mantel-Cox) test. ns, no significance; *, *P* < 0.05; **, *P* < 0.01; ****, *P* < 0.0001.

Prior to the challenge study, we determined that LD_50_ (50% lethal dose) of WT PA103 was 2 × 10^5^ CFU in BALB/c mice by i.n. administration, which was similar to a previous report (31, 32). Groups of mice (*n* = 10 to 15, nearly equal males and females) were immunized intramuscularly with 100 μl PBS containing 50 μg of OMVs from YptbS44(pSMV81) designated rOMV-PH as an experimental group, 50 μg of OMVs from YptbS44(pYA3493), designated rOMV-N, 10 μg of PH-alhydrogel, and PBS-alhydrogel as control groups and then boosted at 21 days after the prime immunization (Fig. 2A). Either rOMV-PH or rOMV-N immunization led to moderate swelling at the injection site a week after injection and anorexia in 2 days postadministration (observation data) and retarded mouse weight gain in the first week after immunization compared to other immunization groups (Fig. 2B) but did not cause obvious health issues in mice. On day 42 after the initial vaccination, the mice were challenged by i.n. administration. The rOMV-PH vaccination afforded 73% protection for mice against i.n. challenge with 5 × 10^6^ (25 LD_50_) of PA103 (Fig. 2C). None of the rOMV-N- or PBS-immunized mice and only 20% of PH-immunized mice survived the same challenge (Fig. 2C).

The PA103 strain producing a 70-kDa cytotoxic protein (ExoU) encoded by *exoU*, exhibits a cytotoxic phenotype, whereas the PAO1 strain manifests an invasive but noncytotoxic phenotype due to lack of ExoU (33). Additionally, the serotypes of PA103 (O11) and PAO1 (O5) are different (34, 35). Further, we tested whether the rOMV-PH vaccination could provide cross-protection against lethal infection of the PAO1 strain. Results demonstrated that the rOMV-PH vaccination afforded complete protection against pulmonary PAO1 infection, while the rOMV-N and PH vaccination provided 20% and 40% protection against the same challenge, respectively (Fig. 2D).

### Antibody responses, the opsonophagocytic killing assay, and cytotoxic inhibition assay.

**FIG 3:**
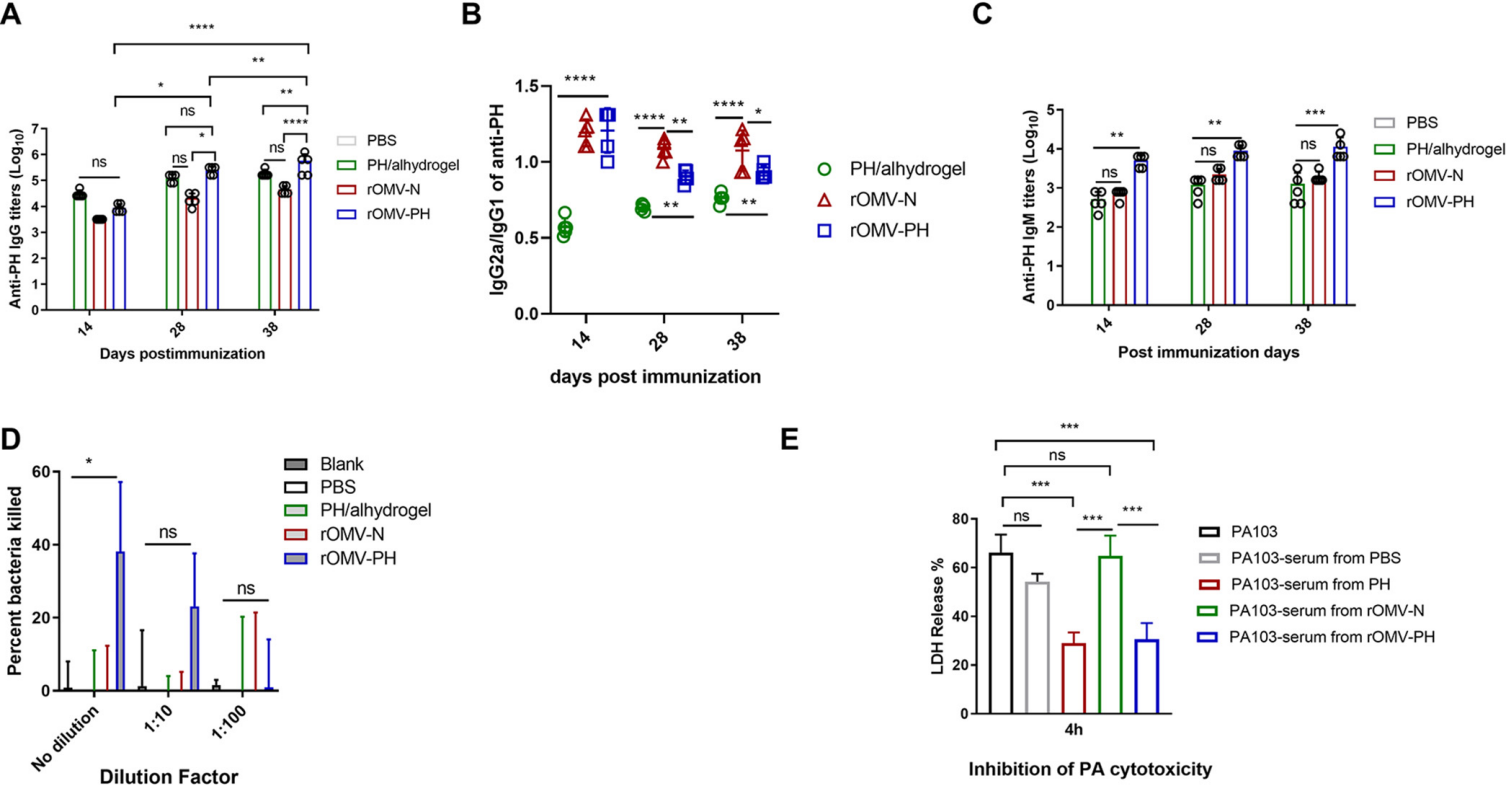
Antibody responses to PH fusion antigen in immunized mice and antibody functional analysis *in vitro*. BALB/c mice were immunized intramuscularly with 50 μg rOMVs–100 μl PBS, 10 μg PH–alhydrogel–100 μl PBS, or alhydrogel alone–100 μl PBS as negative controls and then boosted on day 21 after prime immunization. Blood was collected on days 14, 28, and 38, and antigen-specific antibodies were determined by ELISA. The data represent 10 mice per group. (A) Anti-PH total IgG titers at days 14, 28, and 38 in different immunized mice. (B) Ratios of IgG2a/IgG1 to the PH fusion antigen at days 14, 28, and 38. (C) Anti-PH IgM titers at days 14, 28, and 38 in different immunized mice. (D) Comparative analysis of opsonophagocytic killing activity against PA103 using antisera from different immunized mice. (E) Assay of antibody inhibition of PA103 cytotoxicity to HeLa cells. Sera collected from different immunized mice were used for this assay (see Materials and Methods). Data were shown as the means ± standard deviations (SD). The statistical significance among groups was analyzed by two-way multivariate ANOVA with a Tukey *post hoc* test. ns, no significance; *, *P* < 0.05; **, *P* < 0.01; ***, *P* < 0.001; ****, *P* < 0.0001.

Serum antibody responses showed that both PH- and rOMV-PH prime immunization generated similarly high anti-PH IgG titers in mice 14 and 28 days postvaccination (dpv). Only the rOMV-PH boost immunization substantially increased anti-PH IgG titers (Fig. 3A). Although no PH fusion antigen was present in rOMV-N, the rOMV-N immunization stimulated comparable amounts of unspecific anti-PH IgG titers to PH immunization on 14, 28, and 38 dpv (Fig. 3A). Generally, IgG1 is associated with a Th2-like response while IgG2a is associated with a Th1-like response in mice (36). Therefore, IgG subtypes to the specific antigen produced in immunized mice can distinguish Th1 and Th2 immune responses. In Fig. 3B, both anti-PH IgG2a/IgG1 ratios in the rOMV-PH-immunized group (1.207, 0.903, and 0.930) and the rOMV-N-immunized group (1.191, 1.085, and 1.074) on 14, 28, and 38 dpv were substantially higher than those in the PH-immunized group (0.571, 0.701, and 0.762). Our results demonstrated that the rOMV immunization induced a balanced Th1/Th2 response, while the PH immunization skewed to a Th2-biased response. In addition, the prime-boost rOMV-PH immunization stimulated higher anti-PH IgM titers in mice on 14, 28, and 38 dpv than the PH or rOMV-N-immunization. In contrast, boost rOMV-PH immunization did not enhance anti-PH IgM titers compared to prime immunization (Fig. 3C). Anti-PH IgM titers in the PH- or the rOMV-N-immunized mice were comparable and remained consistent on 14, 28, and 38 dpv (Fig. 3C).

Opsonophagocytic killing (OPK) assay was used to evaluate the correlation of functional antibody levels in serum samples with protection (37, 38). Thus, we measured whether the PH-specific antibodies were protective using an OPK assay. Results showed that only undiluted antisera from rOMV-PH-immunized mice exhibited moderate OPK activity compared with those from PBS-, PH-, and rOMV-N-immunized mice. None of the diluted antisera (1:10 or 1:100) from immunized groups displayed OPK activity (Fig. 3D). Further, sera from immunized mice blocking the PA103 cytotoxicity to HeLa cells were determined. Sera from rOMV-N-immunized mice and PBS control mice failed to provide protection against PA103 cytotoxicity compared to sera from PH- and OMV-PH-immunized mice (Fig. 3E). Sera from PH- and OMV-PH-immunized mice afforded comparable protection against PA103 cytotoxicity (Fig. 3E). Our results imply that the PH-specific antibody can mitigate P. aeruginosa cytotoxicity, but blocking P. aeruginosa cytotoxicity alone is not sufficient to prevent pneumonic infection of P. aeruginosa.

### Vaccination with rOMV-PH induced potent cellular immune responses.

**FIG 4:**
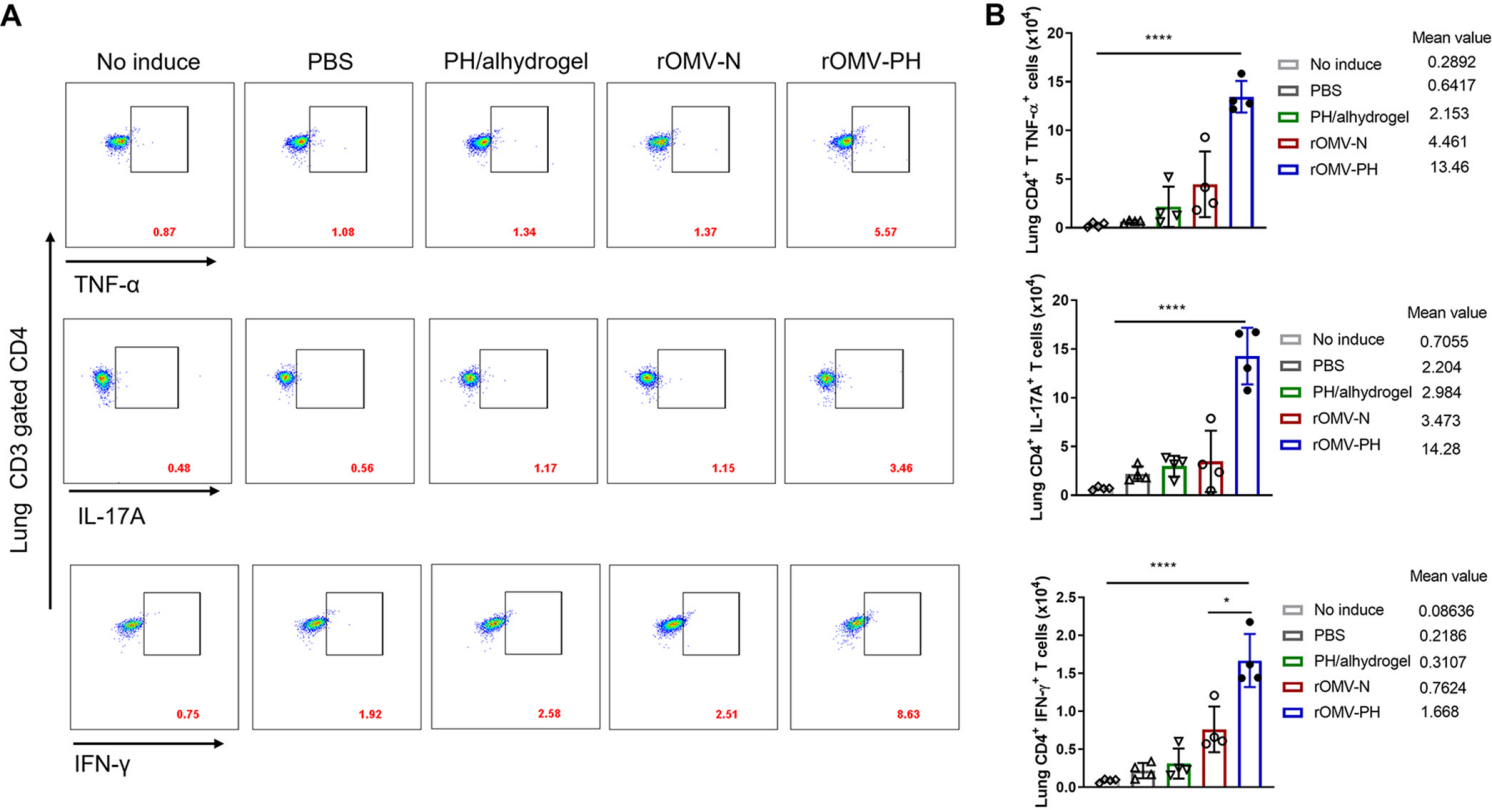
Analysis of lung T-cell responses to the PH stimulation and their cytokines. On day 42 after the initial immunization, lung cells were aseptically isolated from mice (*n* = 4) and stimulated *in vitro* with 10 μg/ml purified recombinant PH-His protein for 48 h to detect specific CD4^+^ and CD8^+^ T cells producing IFN-γ, IL-17, and TNF-α. Cells from PBS-immunized mice without antigen stimulation were used as negative controls. (A) Representative flow cytometry profiles of CD4^+^ T cells producing IFN-γ, IL-17, or TNF-α in the lungs of different immunized mice. (B) Quantification of CD4^+^ IFN-γ^+^-, CD4^+^ IL-17^+^-, and CD4^+^ TNF-α^+^-positive cell numbers in the lungs of mice. Each symbol represents a data point obtained from an individual mouse, with means ± SD. The experiments were performed twice, and data were combined for analysis. The statistical significance among the groups was analyzed by two-way multivariate ANOVA with a Tukey *post hoc* test. ns, no significance; *, *P* < 0.05; **, *P* < 0.01; ***, *P* < 0.001; ****, *P* < 0.0001.

**FIG 5:**
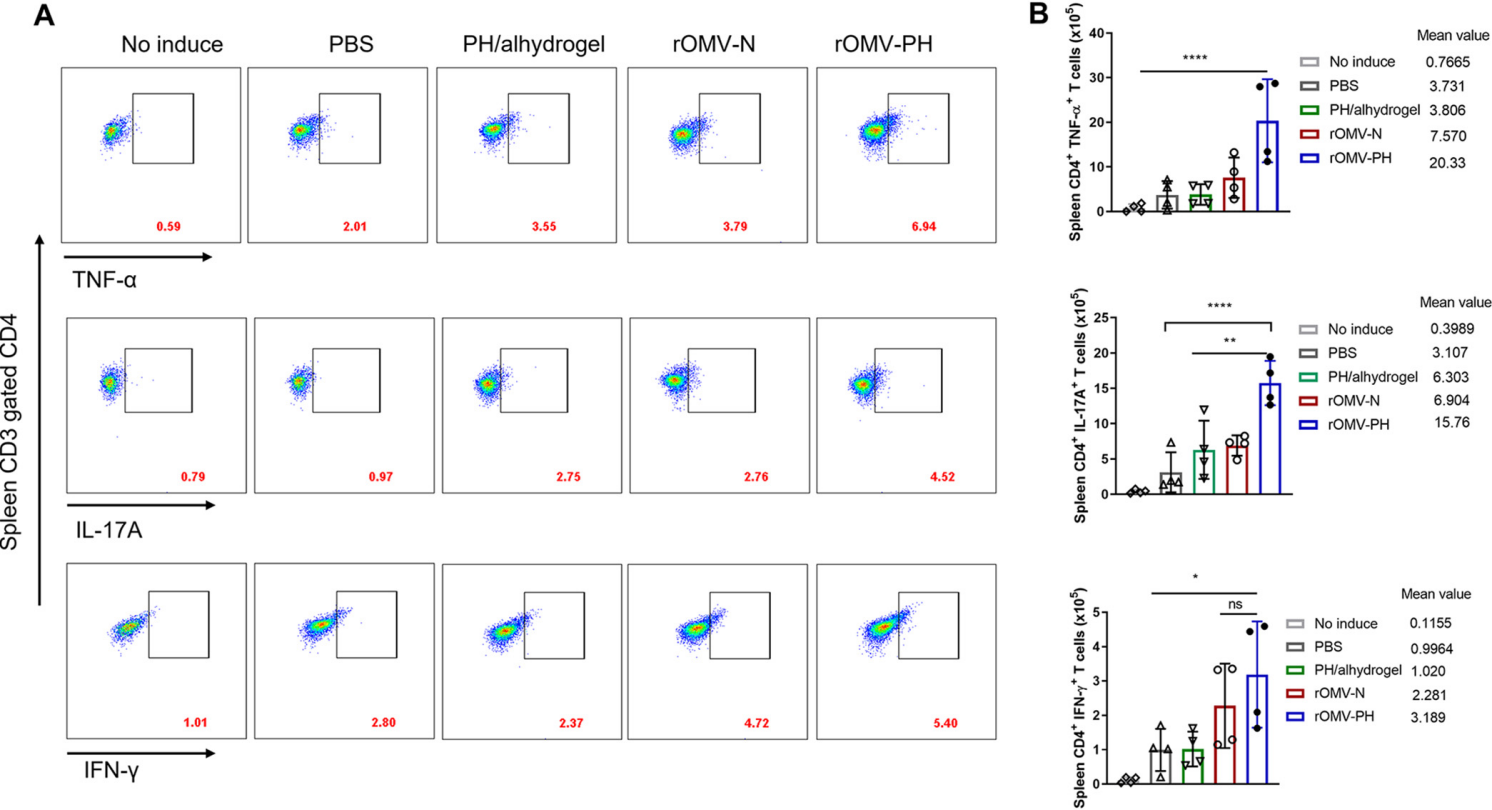
Analysis of spleen T-cell responses to PH stimulation and their cytokines. On day 42 after the initial immunization, splenic cells were aseptically isolated from mice (*n* = 4) and stimulated *in vitro* with 10 μg/ml purified recombinant PH-His protein for 48 h to detect specific CD4^+^ and CD8^+^ T cells encoding IFN-γ, IL-17, and TNF-α. Cells from PBS-immunized mice without antigen stimulation were used as negative controls. (A) Representative flow cytometry profiles of CD4^+^ T cells producing IFN-γ, IL-17, or TNF-α in the spleens of different immunized mice. (B) Quantification of CD4^+^ IFN-γ^+^-, CD4^+^ IL-17^+^-, and CD4^+^ TNF-α^+^-positive cell numbers in the spleens of mice. Each symbol represents a data point obtained from an individual mouse, with means ± SD. The experiments were performed twice, and data were combined for analysis. The statistical significance among the groups was analyzed by two-way multivariate ANOVA with a Tukey *post hoc* test. ns, no significance; *, *P* < 0.05; **, *P* < 0.01; ***, *P* < 0.001; ****, *P* < 0.0001.

We next evaluated T-cell responses in the lung and spleen after immunization. Lung and spleen cells isolated from mice were *in vitro* stimulated with the PH fusion antigen for 48 h and then stained and analyzed using flow cytometry (Fig. 4A and 5A). Quantitative analysis showed that the number of lung CD4^+^ T cells from rOMV-PH-immunized mice producing tumor necrosis factor alpha (TNF-α), interleukin-17A (IL-17A), and gamma interferon (IFN-γ) was significantly higher than that from rOMV-N-, PH-, or PBS-immunized mice (Fig. 4B). In addition, the amounts of TNF-α and IFN-γ produced by lung CD8^+^ T cells from rOMV-PH-, rOMV-N-, or PH-immunized mice were comparable but higher than those from control animals (Fig. S2). Among all groups, there were no significant differences in lung CD8^+^ T cells producing IL-17A (Fig. S2).

10.1128/mSphere.00699-21.2 FIG S2 Analysis of lung CD8^+^ T-cell responses after *in vitro* PH stimulation. On day 42 after the initial immunization, lung cells were aseptically isolated from mice (*n* = 4) and stimulated *in vitro* with 10 μg/ml purified recombinant PH-His protein for 48 h to detect antigen-specific T-cell replication. Cells from PBS-immunized mice without antigen stimulation were used as negative controls. (A) Representative flow cytometry profiles of CD8^+^ T cells producing IFN-γ, IL-17, or TNF-α in the lungs of different immunized mice. (B) Quantification of CD8^+^ IFN-γ^+^-, CD8^+^ IL-17^+^-, and CD8^+^ TNF-α^+^-positive cell numbers in the lungs of mice. Each symbol represents a data point obtained from an individual mouse, with means ± SD. The experiments were performed twice, and data were combined for analysis. The statistical significance among the groups was analyzed by two-way multivariate ANOVA with a Tukey *post hoc* test. ns, no significance; *, *P* < 0.05; **, *P* < 0.01; ***, *P* < 0.001; ****, *P* < 0.0001. Download FIG S2, PDF file, 0.1 MB. Copyright © 2021 Li et al. 2021 Li et al. https://creativecommons.org/licenses/by/4.0/ This content is distributed under the terms of the Creative Commons Attribution 4.0 International license.

After PH stimulation, spleen CD4^+^ T cells from rOMV-PH-immunized mice producing TNF-α and IL-17A were significantly higher than those from rOMV-N-, PH-, or PBS-immunized mice (Fig. 5B). Spleen CD4^+^ IFN-γ-producing cells from rOMV-PH- or rOMV-N-immunized mice were comparable but significantly higher than those from PH- or PBS-immunized mice (Fig. 5B). Similarly, spleen CD8^+^ T cells from rOMV-PH-immunized mice produced larger amounts of IFN-γ and TNF-α than those from rOMV-N-, PH-, or PBS-immunized mice (Fig. S3). There were also no significant differences in spleen CD8^+^ T cells producing IL-17A among all groups of animals (Fig. S3). Altogether, the rOMV-PH vaccination induced potent PH-specific Th1 and Th17 responses in the lungs and spleens of mice compared to the other vaccinations.

10.1128/mSphere.00699-21.3 FIG S3 Analysis of spleen CD8^+^ T-cell responses after *in vitro* PH stimulation. On day 42 after the initial immunization, splenic cells were aseptically isolated from mice (*n* = 4) and stimulated *in vitro* with 10 μg/ml purified recombinant PH-His protein for 48 h to detect antigen-specific T-cell replication. Cells from PBS-immunized mice without antigen stimulation were used as negative controls. (A) Representative flow cytometry profiles of CD8^+^ T cells producing IFN-γ, IL-17, or TNF-α in the spleens of different immunized mice. (B) Quantification of CD8^+^ IFN-γ^+^-, CD8^+^ IL-17^+^-, and CD8^+^ TNF-α^+^-positive cell numbers in the spleens of mice. Each symbol represents a data point obtained from an individual mouse, with means ± SD. The experiments were performed twice, and data were combined for analysis. The statistical significance among the groups was analyzed by two-way multivariate ANOVA with a Tukey *post hoc* test. ns, no significance; *, *P* < 0.05; **, *P* < 0.01; ***, *P* < 0.001; ****, *P* < 0.0001. Download FIG S3, PDF file, 0.1 MB. Copyright © 2021 Li et al. 2021 Li et al. https://creativecommons.org/licenses/by/4.0/ This content is distributed under the terms of the Creative Commons Attribution 4.0 International license.

### The rOMV-PH vaccination effectively controlled bacteria and host inflammation.

**FIG 6:**
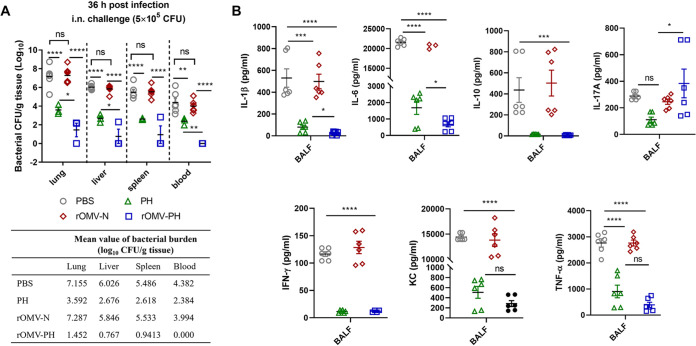
*In vivo* responses after i.n. challenge with P. aeruginosa PA103. PBS-, PH-, rOMV-N-, or rOMV-PH-immunized BALB/c mice (*n* = 6) were infected i.n. with a lethal dose (5 × 10^5^ CFU) of PA103. On 36 h postchallenge, different tissues (lung, liver spleen, and blood) were collected from euthanized mice. (A) Bacterial burden was evaluated in the lungs, livers, spleens, and blood. (B) Amounts of cytokine/chemokine (IL-1β, IL-6, IL-10, IL-17A, IFN-γ, KC, and TNF-α) in bronchoalveolar lavage fluid (BALF) from immunized mice 36 h postinfection. Data are shown as the means ± SD. The experiments were performed twice, and data were combined for analysis. The statistical significance among the groups was analyzed by two-way multivariate ANOVA with a Tukey *post hoc* test. ns, no significance; *, *P* < 0.05; **, *P* < 0.01; ***, *P* < 0.001; ****, *P* < 0.0001.

To evaluate *in vivo* responses of immunized mice, mice were challenged with 5 × 10^5^ CFU PA103 by i.n. administration. Bacterial burdens in different tissues and cytokine/chemokine production in bronchoalveolar lavage fluid (BALF) were analyzed. At 36 h postinfection, PBS- or rOMV-N-immunized mice had strikingly increased bacterial titers in the lungs (mean value, 7.155 or 7.287 log_10_ CFU/g tissue). Bacteria also rapidly disseminated to the livers (mean value, 6.026 or 5.846 log_10_ CFU/g tissue), the spleens (mean value, 5.486 or 5.533 log_10_ CFU/g tissue), and the blood (mean value, 4.382 or 3.994 log_10_ CFU/g tissue). Compared to the PBS immunization, the PH or OMV-PH immunization substantially decreased bacterial burdens within the lung, liver, and spleen of mice. However, the rOMV-PH immunization had more efficiency to clear bacteria from mouse blood than the PH immunization (Fig. 6A). After 72 h of infection, no bacteria were detected in organs of the rOMV-PH-immunized mice (Fig. S4B). Analysis of BALF cytokine/chemokine in mice after i.n. infection showed that dramatically high levels of cytokines (IL-1β, IL-6, IL-10, IFN-γ, and TNF-α) and chemokine (KC) were secreted into the BALF of PBS- or rOMV-N-immunized mice 36 h postinfection compared to those in PH- or rOMV-PH-immunized mice. The rOMV-PH-immunized mice produced smaller amounts of IL-1β and IL-6 than the PH-immunized mice did (Fig. 6B).

10.1128/mSphere.00699-21.4 FIG S4 T-cell depletion and bacterial burden in rOMV-PH-immunized mice after 72 h postinfection. (A) A representative scatter-plot graph showing depletion of CD4^+^ and CD8^+^ T cells by i.p. injection of mouse anti-CD4, anti-CD8, and both monoclonal antibodies. (B) PBS-, PH-, rOMV-N, or rOMV-PH-immunized BALB/c mice (*n* = 3) were infected i.n. with a lethal dose (5 × 10^5^ CFU) of PA103. At 72 h postchallenge, different tissues (lung, liver, spleen, and blood) were collected from euthanized mice and homogenized, and then 100-μl samples of 1 ml of undiluted tissue homogenates were spread on LB agar plates for bacterial enumeration. (C) BALB/c mice (*n* = 5) were passively immunized with 100 μl of sera collected from mice immunized with rOMVs, PH, or PBS. Blood was collected on day 1 postadministration for serum isolation, and serum anti-PH antibody was measured by ELISA. The data represent 5 mice per group. Each symbol represents a data point obtained from an individual mouse, with means ± SD. The statistical significance among the groups was analyzed by two-way multivariate ANOVA with a Tukey *post hoc* test. ns, no significance; *, *P* < 0.05; **, *P* < 0.01; ***, *P* < 0.001; ****, *P* < 0.0001. Download FIG S4, PDF file, 0.1 MB. Copyright © 2021 Li et al. 2021 Li et al. https://creativecommons.org/licenses/by/4.0/ This content is distributed under the terms of the Creative Commons Attribution 4.0 International license.

### T cells, but not antibodies, are essential for protection against pulmonary P. aeruginosa infection.

**FIG 7:**
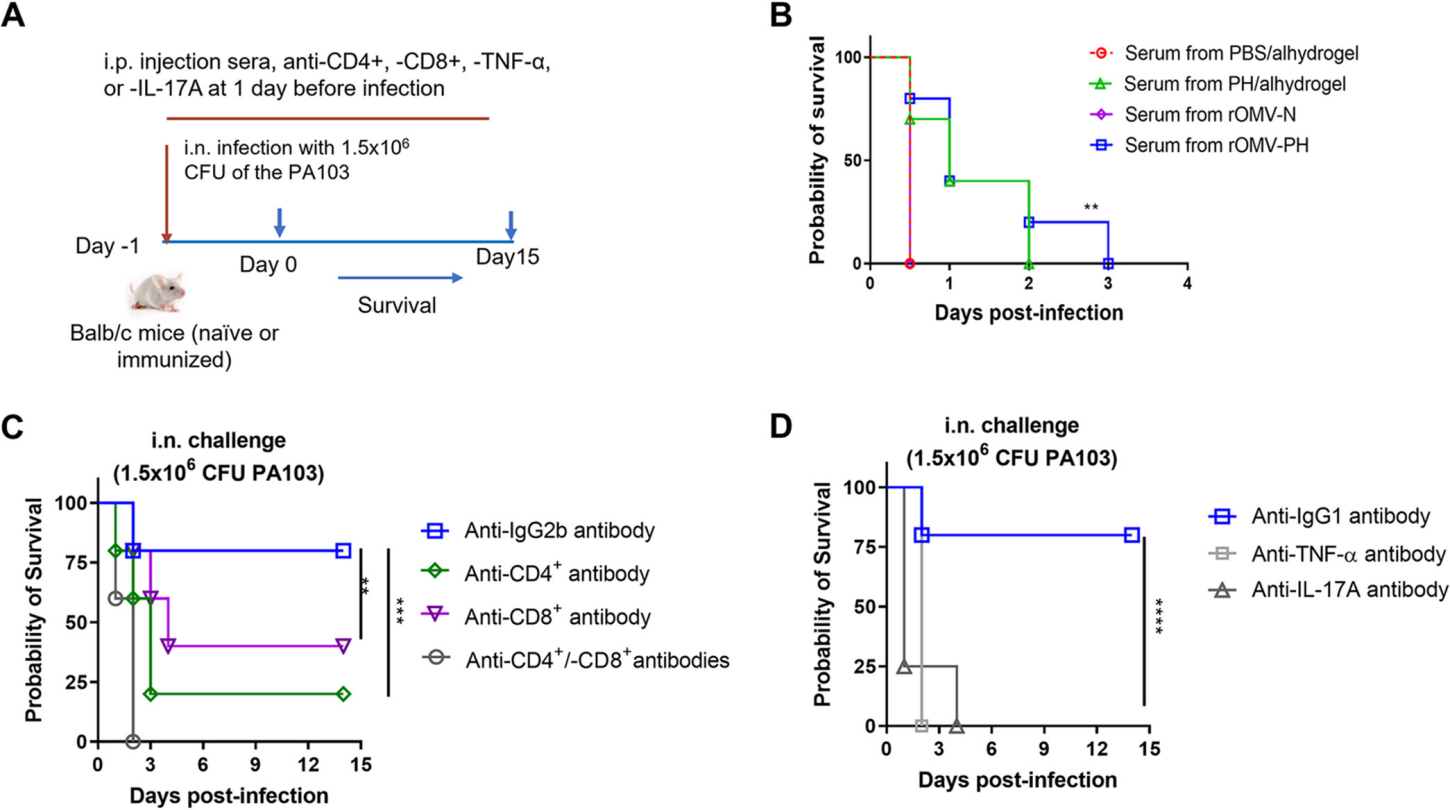
Roles of antibodies, T cells, and cytokines (TNF-α and IL-17A) in protection against i.n. challenge with P. aeruginosa PA103. (A) Scheme of serum transfer, T-cell depletion, and neutralization of TNF-α or IL-17A. (B) Serum transfer. Protection against pneumonic P. aeruginosa infection from transferred sera. Naïve BALB/c mice (*n* = 10) were intraperitoneally injected with 100 μl of sera collected from PBS-, PH-, rOMV-, or rOMV-PH-immunized mice 38 dpv. Twenty-four hours postinjection, recipient mice were i.n. challenged with 1.5 × 10^6^ CFU of the PA103 strain. (C) T-cell depletion. The rOMV-PH-immunized mice (*n* = 5) were depleted of CD4^+^ and/or CD8^+^ T cells by intraperitoneal administration with 500 μg of anti-CD4^+^, anti-CD8^+^, and both MAbs. The isotype-matched rat IgG2b MAb (clone LTF-2) was used as a control. Twenty-four hours postinjection, mice were intranasally challenged with 1.5 × 10^6^ CFU of the PA103 strain. (D) Neutralization of cytokine. The rOMV-PH immunized mice (*n* = 5) were intraperitoneally injected with 200 μg of anti-TNF-α or anti-IL-17A MAbs. The isotype-matched rat IgG1 MAb (clone HRPN) was used as a control. Twenty-four hours postinjection, mice were intranasally challenged with 1.5 × 10^6^ CFU of the PA103 strain. Statistical significance was analyzed by log-rank (Mantel-Cox) test. ns, no significance; *, *P* < 0.05; **, *P* < 0.01, ****, *P* < 0.0001.

To further determine the protective roles of antibodies and T cells, we performed experiments of passive transfer of immune serum, T-cell depletion, and cytokine neutralization (Fig. 7A). Sera collected from rOMV-PH-, rOMV-N-, or PH-immunized mice were passively transferred to groups of naive mice on day 1 postinjection. Antibody analysis showed that titers of anti-PH IgG in the recipient mice were 10-fold lower than those in rOMV-PH- or PH-immunized mice (Fig. 3A, Fig. S4C). The passive immunization failed to provide any protection against pneumonic P. aeruginosa infection but could extend their death time (Fig. 7B). Compared to injection with the isotype control (rat IgG2b monoclonal antibody [MAb]), rOMV-PH-immunized mice (*n* = 5) depleted of CD4^+^ T cells only had 20% survival, depleted of CD8^+^ T cells had 40% survival, and depleted of both CD4^+^ and CD8^+^ T cells completely lost protection against i.n. challenge with P. aeruginosa (Fig. 7C). Both lung and spleen T cells produced significant amounts of TNF-α and IL-17A after stimulation with PH antigen (Fig. 4 and 5), suggesting that TNF-α and IL-17A are important for pneumonic P. aeruginosa infection. Compared to injection with the isotype control (rat IgG1 MAb), individual neutralization of either TNF-α or IL-17A in rOMV-PH-immunized mice (*n* = 5) led to the loss of protection against i.n. challenge with P. aeruginosa (Fig. 7D). Our results highlighted that T cells and corresponding cytokines (TNF-α or IL-17A) play critical roles in protection against pneumonic P. aeruginosa infection.

## DISCUSSION

The increasing prevalence of multidrug-resistant P. aeruginosa infection in health care settings justifies the urgent need for an effective vaccine against this organism. Barriers to P. aeruginosa vaccine development include the presence of phenotypically diverse P. aeruginosa strains, the diverse virulence mechanisms, and the lack of reliable animal models to mimic CF patients (39, 40). Human clinical trials showed that the anti-PcrV antibody or its fragment could reduce inflammation and damage in the airway of CF patients (41). However, directly using PcrV antigen as a vaccine component has not been evaluated in human clinical trials yet, due to protein purity (42) or other reasons. In addition, Holder et al. reported that immunization with PcrV alone did not provide long-term protection to the burned mice infected with the highly toxigenic strain 1071 (24). Moreover, purified antigens as subunit vaccines administered alone have limited immunogenicity (43), and vaccination with subunit vaccines is prone to generating a humoral response (44). Many previous studies have illustrated that an excellent P. aeruginosa vaccine should stimulate humoral and Th1/Th17-type CD4^+^ T-cell responses to provide effective protection against pulmonary and systemic P. aeruginosa infection (42, 45, 46). Currently, the dilemma for subunit vaccines is being addressed with different improved vaccine carriers (47). Among them, using self-adjuvanting OMVs as a carrier not only circumvents the requirements of antigen purification for traditional subunit vaccines but also stimulates potent specific humoral and cellular responses to the delivered antigens (15, 21).

Our studies showed that i.m. immunization with rOMV-PH afforded 73% protection against i.n. challenge with the cytotoxic PA103 strain (Fig. 2C) and complete protection against i.n. challenge with the noncytotoxic strain PAO1 (Fig. 2D), which provides evidence that using rOMVs from a recombinant Y. pseudotuberculosis strain to deliver a heterologous PH fusion antigen of P. aeruginosa was feasible. Thus, a combination of triple or tetra-antigen may further enhance protection against P. aeruginosa infection. In addition, current OMVs from YptbS44(pSMV81) contained mixed MPLA and hexa-acylated lipid A (endotoxin) due to incomplete modification by LpxE. Immunization with rOMV-N or rOMV-PH caused ∼10% weight loss in mice due to endotoxin and other uncharacterized components that elicited high inflammation and reactogenicity. Thus, further studies will be pursued to genetically modify YptbS44 to uniformly synthesize detoxified tetra-acylated lipid A (48, 49), eliminate potential virulence factors, and express multiple P. aeruginosa antigens, which improves immunogenicity but reduces toxicity of OMVs.

Generally, antigen-specific antibodies induced by a vaccine candidate can correlate with protection and assist in the killing of host target cells infected by bacteria (50). However, our results showed that immunization with PH-alhydrogel generated titers of PH-specific antibodies in mice comparable to those of the rOMV-PH immunization (Fig. 3A) but failed to provide effective protection against i.n. challenge with the PA103 or PAO1 strain (Fig. 2C and D). A possible reason for this the PH immunization-stimulated Th2-biased immune response, while rOMV-PH immunization induced a balanced Th1/Th2 immune response (Fig. 3B). Another reason is that rOMV-PH immunization induced significantly higher anti-PH IgM titers in mice than PH immunization (Fig. 3C). Racine and Winslow indicated that IgM provides the first line of defense during microbial infections before the generation of high-affinity IgG responses (51). However, whether the antigen-specific IgM is involved in protection needs to be further investigated. In addition, sera from rOMV-N-immunized mice presented high nonspecific anti-PH IgG and IgM titers and were comparable to sera from PH-immunized mice (Fig. 3A and C). One possible explanation for this is that the purified PH fusion antigen, as the enzyme-linked immunosorbent assay (ELISA) coating protein contains other protein components and smaller amounts of endotoxin (Fig. S1E) that can be recognized by sera from rOMV-N-immunized mice. Another explanation is that the antiserum to certain components of the Yptb OMVs have cross-reactivity to the PH fusion antigen, for example, protein alignment indicates that a periplasmic iron(III)-binding protein (YPTS_2780) in Yptb has 41% identity with HitA. However, the high background antibody responses in the rOMV-N-immunized mice did not provide any protection against pneumonic P. aeruginosa infection (Fig. 2C), which suggests that cellular immune responses are critical to protection against pneumonic P. aeruginosa infection. Intriguingly, *in vitro* OPK assay showed that sera from rOMV-PH-immunized mice could kill P. aeruginosa to a certain extent instead of sera from PH-immunized mice (Fig. 3D). Our results were not consistent with several previous studies, in which anti-PcrV_NH_ sera from the PcrV_NH_-immunized mice (52) or POH (PcrV-OprI-Hcp1)-specific antibodies from the trivalent subunit of POH-vaccinated mice (42) exhibited significant OPK activity to P. aeruginosa. Thus, we speculate that distinct antigen combinations or different antibody compositions in sera from immunized mice lead to the inconsistency.

Measurement of T-cell responses in the lung and spleen showed that the rOMV-PH immunization stimulated substantially higher PH-specific Th1 and Th17 responses than the PH or rOMV-N immunization (Fig. 4 and 5), which is well correlated with animal survival after challenge (Fig. 2C and D). Consistent with the immune protection and response, rOMV-PH-immunized mice could more efficiently clear P. aeruginosa after i.n. challenge than PH-, rOMV-N-, or PBS-immunized mice (Fig. 6A). The activation of the immune system and production of inflammatory cytokines and associated chemokines are essential for the natural antibacterial immune responses (53, 54). However, hyperactivation of these immune responses leads to an acute increase in the secretion of proinflammatory cytokines, resulting in detrimental consequences to hosts (55). Studies have implicated that overproduction of IL-1β, IL-6, TNF-α, and IL-10 in mice and humans is associated with sepsis (56), and IFN-γ expression is enhanced persistently in patients who die of sepsis (57). KC is essential for neutrophil migration and expression of proinflammatory mediators (58); thus, overmounts of KC in sera recruiting excessive neutrophils may cause extensive tissue damage and immune dysregulation. Amounts of IL-1β, IL-6, TNF-α, IFN-γ, IL-10, and KC secreted into BALF samples of PBS- or rOMV-immunized mice after i.n. challenge with PA103 were significantly higher than those of rOMV-PH- or PH-immunized mice (Fig. 6B). Significant increases of IL-1β and IL-6, and slight elevation of TNF-α and KC in BALF samples of PH-immunized mice after infection, were observed compared to those of rOMV-PH-immunized mice (Fig. 6B), implying that the rOMV-PH immunization more effectively coordinated cytokine/chemokine production in mice by rapidly eliminating bacterial burden in organs than the PH, rOMV-N, or PBS immunization. However, upon P. aeruginosa infection, excessive production of proinflammatory cytokines induced by high bacterial burden in organs in PH-, rOMV-N-, or PBS-immunized mice are detrimental instead of protective. In addition, rOMV-PH-immunized mice produced substantially higher IL-17 in BALFs than PH-, rOMV-N-, or PBS-immunized mice after i.n. P. aeruginosa challenge (Fig. 6B), suggesting that IL-17 plays a protective role against P. aeruginosa infection.

Previous studies have evidenced that TNF-α plays an important role in P. aeruginosa clearance and resistance to P. aeruginosa infection (59, 60), and Th17 cells producing IL-17A confer protection against pneumonic P. aeruginosa infection (45, 61, 62). Consistent with those studies, depletion of T cells, and neutralization of cytokines (TNF-α or IL-17A), underlined that T cells, TNF-α, and IL-17A all were critical for comprehensive protection against pneumonic P. aeruginosa infection (Fig. 7C and D). Since TNF-α is a double-edged factor for regulating immune responses (63), appropriate amounts of TNF-α secretion can promote host defense, whereas its excess production is lethal. In the rOMV-PH-immunized animals, memory T cells or other cells promptly secret TNF-α and other cytokines upon P. aeruginosa infection, rapidly clear PA in the lung, and help the lung return to homeostasis. However, excessive production of TNF-α or other proinflammatory cytokines in the lungs of PBS-, rOMV-N-, or PH-immunized animals will result in immune dysregulation, leading to ineffectively clearing bacteria, loss of lung function, and eventually death. Intriguingly, Sen-Kilic et al. recently highlighted the importance of the humoral immune response for protection against P. aeruginosa infection and showed that passive immunization with serum from mice vaccinated with a curdlan-adjuvanted P. aeruginosa whole-cell vaccine (WCV) alone protected naive mice against P. aeruginosa (64). However, our results in antibody passive immunization and T-cell depletion were inconsistent with their observations. The explanations for inconsistencies may be because the type of vaccine, P. aeruginosa challenge strain, and mouse strain used in the two studies are different. In addition, our results showed that small amounts of anti-PH antibody in recipient mice extended their death time. Thus, we speculate that transferring large amounts of anti-PH serum from OMV-PH- or PH-immunized mice provide partial protection. The detailed roles of B cells, T cells, TNF-α, and IL-17A induced by the rOMV-PH immunization for protection need to be pursued further. Altogether, as a proof-of-concept study, OMVs delivering heterologous Pseudomonas antigens would be a novel type of vaccine restraining the spread of P. aeruginosa in health care settings.

## MATERIALS AND METHODS

### Bacterial strains, plasmids, culture conditions, and molecular operations.

All bacterial strains and plasmids used in this study are listed in Table 1. All bacterial cultures and molecular and genetic procedures used in this study are described in Text S1 in the supplemental material.

### Isolation and analysis of lipid A and OMVs.

The detailed procedures for isolation and analysis of lipid A from Yptb and its OMVs were performed as described previously (65, 66), with minor modifications (Text S1). Isolation of OMVs from Y. pseudotuberculosis strains was similar to that described previously (30). A brief procedure was described in Text S1. The OMVs were analyzed by transmission electron microscopy (TEM), and a Bradford assay was performed for quantifying the total protein abundance associated with OMVs as described previously (30). The heterologous antigen present in the OMV preparations was detected by immunoblotting.

### Protein purification.

Escherichia coli TOP10 carrying pSMV82 (*pcrV-hitA_T_*-6×His) was grown overnight at 37°C in LB broth. Full-length *his*-tagged *pcrV* was expressed from E. coli χ6212(pSMV67). The procedure for protein purification by nickel chromatography is described previously (67). To remove the remaining endotoxin, the purified PcrV-HitA_T_ (PH) protein was passed through Pierce high-capacity endotoxin removal spin columns (ThermoFisher Scientific). Ten micrograms of His-tagged PcrV protein was emulsified with alhydrogel adjuvant and injected into BALB/c mice. Mice were then immunized with two booster injections at 3-week intervals. Sera were collected 1 week after the last booster injection.

### Animal experiments.

Animal protocols followed the NIH *Guide for the Care and Use of the Laboratory Animals* (68) and were approved by the Institutional Animal Care and Use Committee at Albany Medical College (IACUC protocol number 20-02001). Six-week-old male and female BALB/c mice were purchased from Taconic (Germantown, NY) and acclimated for 1 week after arrival. Mice were primed by intramuscular (i.m.) vaccination and then boosted at 3 weeks after the initial vaccination. Blood samples were collected via submandibular veins at 2-week intervals to harvest sera for antibody analysis. On 42 days after the initial vaccination, animals were anesthetized with a 1:5 xylazine-ketamine mixture and challenged intranasally (i.n.) with a lethal dose of a P. aeruginosa strain in 40 μl PBS. All infected animals were observed over 15 days. The actual numbers of bacterial CFU were determined by plating serial dilutions of the inoculum on LB agar plates.

For the determination of the bacterial burden, infected animals were euthanized with an overdose of sodium pentobarbital. Lung, liver, spleen, and blood were taken at the indicated times and homogenized in ice-cold PBS (pH 7.4) using a bullet blender at power 7 for 2 min. Serial dilutions of each organ homogenate were plated on LB agar plates, and each count was confirmed with duplicate plates with different dilutions to determine the titers of bacteria per gram of tissue. A mouse multiplex cytokine assay kit (Bio-Plex; Bio-Rad) was used to detect cytokines and chemokines in the bronchoalveolar lavage fluid (BALF) collected from the mice according to the manufacturer’s instructions.

### Antibody responses and opsonophagocytic killing assay.

Antibody titers were measured using an ELISA described in Text S1. The opsonophagocytic killing assay was performed as described previously (42). Briefly, HL-60 cells (CCL-240; ATCC) were differentiated into granulocyte-like cells in Iscove's modified Dulbecco's medium (IMDM) (ATCC) containing 100 mM *N*′,*N*-dimethylformamide (Sigma) for 5 days. Serum samples from immunized mice containing opsonic antibodies were heat inactivated (56°C, 30 min) and serially diluted with opsonization buffer (mixture of 80 ml of sterile water, 10 ml of 10× Hanks’ balanced solution, 10 ml of 1% gelatin, and 5.3 ml of fetal bovine serum). Each well in a 96-well plate contained 40 μl of 4 × 10^5^ HL60 cells, 10^3^ CFU of PA103 in 10 μl of opsonophagocytic buffer, 20 μl of serum, and 10 μl of 1% infant rabbit serum as a complement source (Sigma). Blank wells with the same system in the absence of mouse serum were used as negative controls. After 2 h of incubation, 10 μl of each sample was plated on LB agar medium. Each sample was performed in triplicate. The opsonophagocytic killing ability was defined as a reduction in number of CFU compared with the number of CFU in the sera from unimmunized mice.

### Inhibition of P. aeruginosa cytotoxic assay.

Sera from immunized mice for inhibiting cytotoxicity of P. aeruginosa were assayed as described previously (69), with minor modifications. HeLa cells were seeded in 48-well plates (2.5 × 10^4^ cells/well). P. aeruginosa PA103 was grown to log phase and diluted to obtain the desired multiplicity of infection (1:10). The serum was heat inactivated at 56°C for 30 min and incubated with the PA103 for 15 min at room temperature at a dilution of 1:500. HeLa cells were infected with the bacteria that were incubated with different sera or PBS as a control. The plates were incubated at 37°C, 5% CO_2_ for 4 h, and the release of lactate dehydrogenase (LDH) in the supernatant of infected cells was determined using a CytoTox 96 nonradioactive cytotoxicity assay kit (Promega). The percent cytotoxicity was calculated using the formula (experimental − ES − TS + *M*)/(*T*_max_ − TS), where experimental is the optical density at 490 nm (OD_490_) from the well being tested, ES is the OD_490_ from bacteria only, TS is the OD_490_ from untreated cells, *M* is the OD_490_ from media, and *T*_max_ is the OD_490_ from lysed cells (obtained by adding lysis solution 45 min before the end of incubation).

### Analysis of cellular immune responses.

Lungs and spleens were obtained aseptically from euthanized animals. Lungs were minced and digested with 400 μg/ml of Liberase and 30 μg/ml of DNase (Sigma) at 37°C for 30 min. Tissues then were dissociated with 70-μm strainers to obtain single cells. The red blood cell-lysed individual cell populations (2 × 10^6^) were seeded in 12-well cell culture plates and stimulated *in vitro* for 48 h with 10 μg/ml PH. Cells without antigen stimulation were used as negative controls. Four hours before the collection of the cells, cells in each well were supplemented with brefeldin A and a monensin cocktail (1:1 ratio) to block Golgi-mediated cytokine secretion. For the flow-cytometric analysis of the T-cell populations and their corresponding cytokines, the induced cells were harvested and resuspended in fluorescence-activated cell sorting staining buffer containing CD16/32 antibodies (1:200) for 10 min on ice. The T-cell-specific markers were stained using anti-mouse CD3 (fluorescein isothiocyanate), CD4 (phycoerythrin), and CD8 (allophycocyanin [APC]) antibodies (BioLegend, CA), followed by intracellular cytokine (IFN-γ, peridinin chlorophyll protein Cy5.5, TNF-α, BV510, IL17A, and APC-Cy7) staining using BioLegend perm-fix solution and buffer according to the manufacturer’s protocol. The entire staining process was performed on ice with 1 h of incubation at each step. Stained cells (5 × 10^5^) were acquired on BD flow cytometers (FACSymphony A3) and analyzed using FlowJo v.10.

### Antibody transfer, T-cell depletion, and cytokine neutralization.

Eight-week-old naive BALB/c mice were intraperitoneally (i.p.) injected with 100 μl of sera collected at 38 days postvaccination (dpv). Naïve mice received 100 μl of sera from unimmunized mice as controls. At 24 h postadministration, mice were i.n. challenged with a lethal dose of PA103 (1 × 10^6^ CFU). T cells were depleted by i.p. injection with 500 μg of anti-mouse CD4 (clone GK1.5) and/or CD8 (clone 2.43) MAbs initiated 1 day prior to infection and every other day thereafter. Control mice received an equal quantity of isotype-matched rat IgG2b MAb (clone LTF-2). Cytokines were neutralized by i.p. administration of 200 μg of rat IgG1mAb specific for TNF-α (clone XT3.11) or 200 μg of MAb specific for IL-17A (clone 17F3) to each mouse by following the same neutralization strategy as T-cell depletion. Control mice received 200 μg of isotype-matched rat IgG1 MAb (clone HRPN). All MAbs were supplied by Bio X Cell (West Lebanon, NH).

### Statistical analysis.

The statistical analyses of the data among the groups were performed with one-way analysis of variance (ANOVA)/univariate or two-way ANOVA with Tukey *post hoc* tests. The log-rank (Mantel-Cox) test was used for the survival analysis. All data were analyzed using GraphPad PRISM 8.0 software. The data were represented as the means ± standard deviations (SD). ns, no significance; *, *P* < 0.05; **, *P* < 0.01; ***, *P* < 0.001; ****, *P* < 0.0001.
