# Supplementary figures and images for: Outer Membrane Vesicles Displaying a Heterologous PcrV-HitA Fusion Antigen Promote Protection against Pulmonary Pseudomonas aeruginosa Infection
Source: mSphere. 2021 Oct 6;6(5):e00699-21. doi: 10.1128/mSphere.00699-21 (PMC8510544; doi:10.1128/mSphere.00699-21)

A

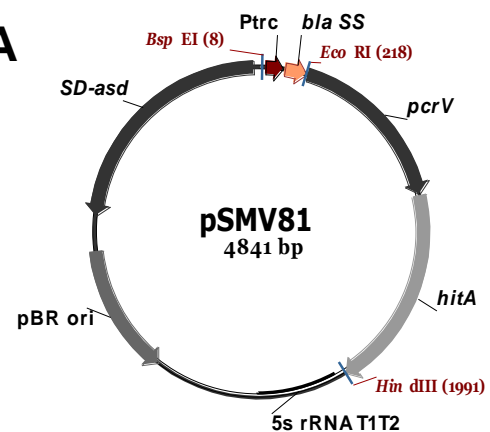

C

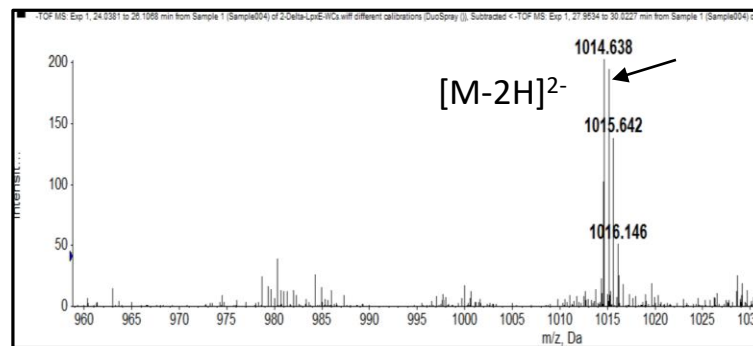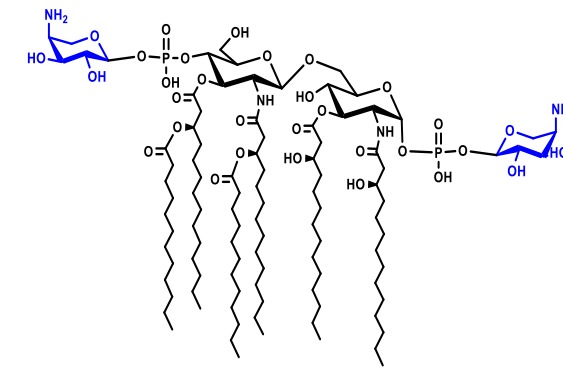

Chemical Formula:  $C_{102}H_{192}N_4O_{31}P_2$   
Exact Mass: 2031.305

B

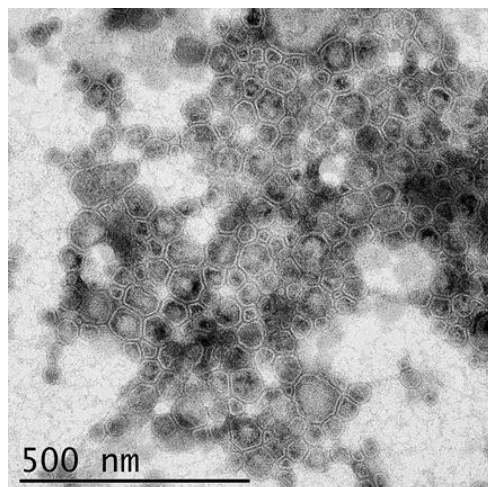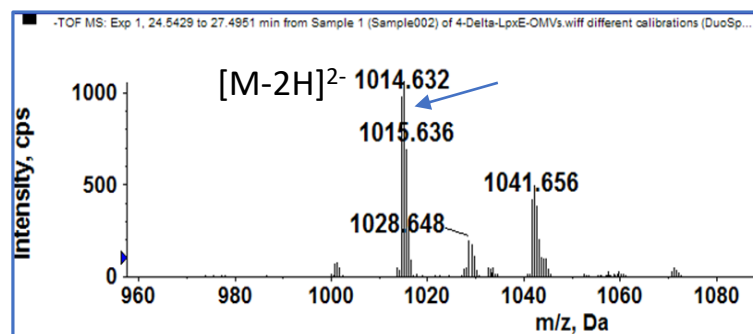

D

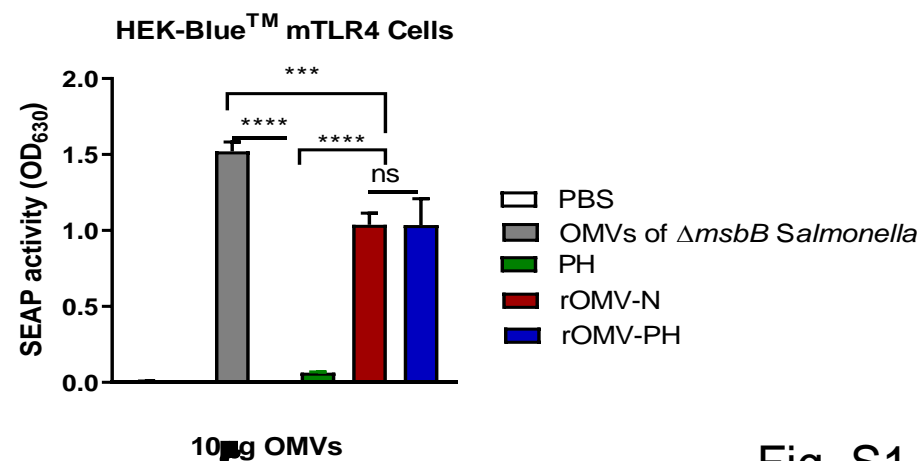

E

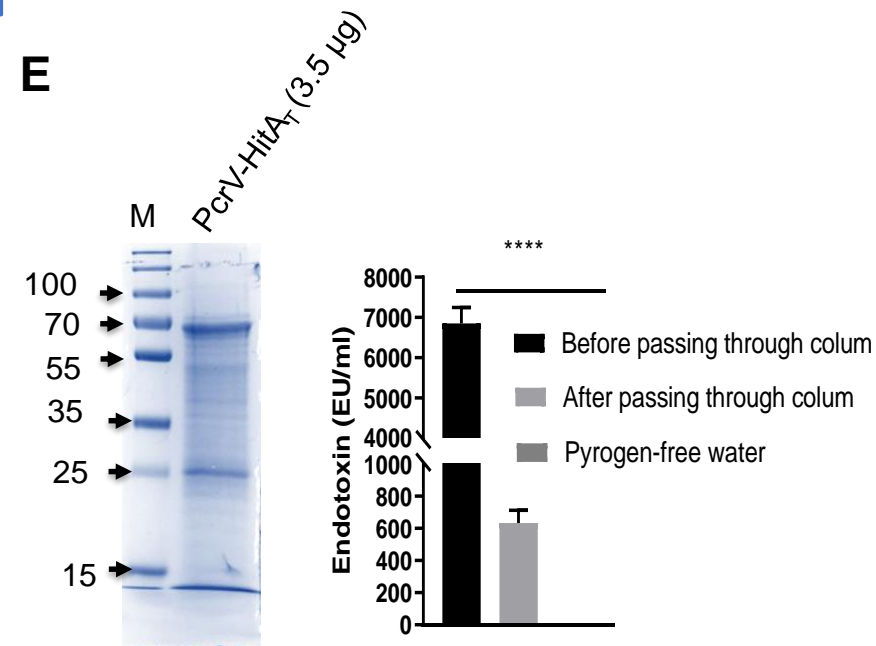

Fig. S1

Supplement: FIG S1 [file msphere.00699-21-sf001.pdf]

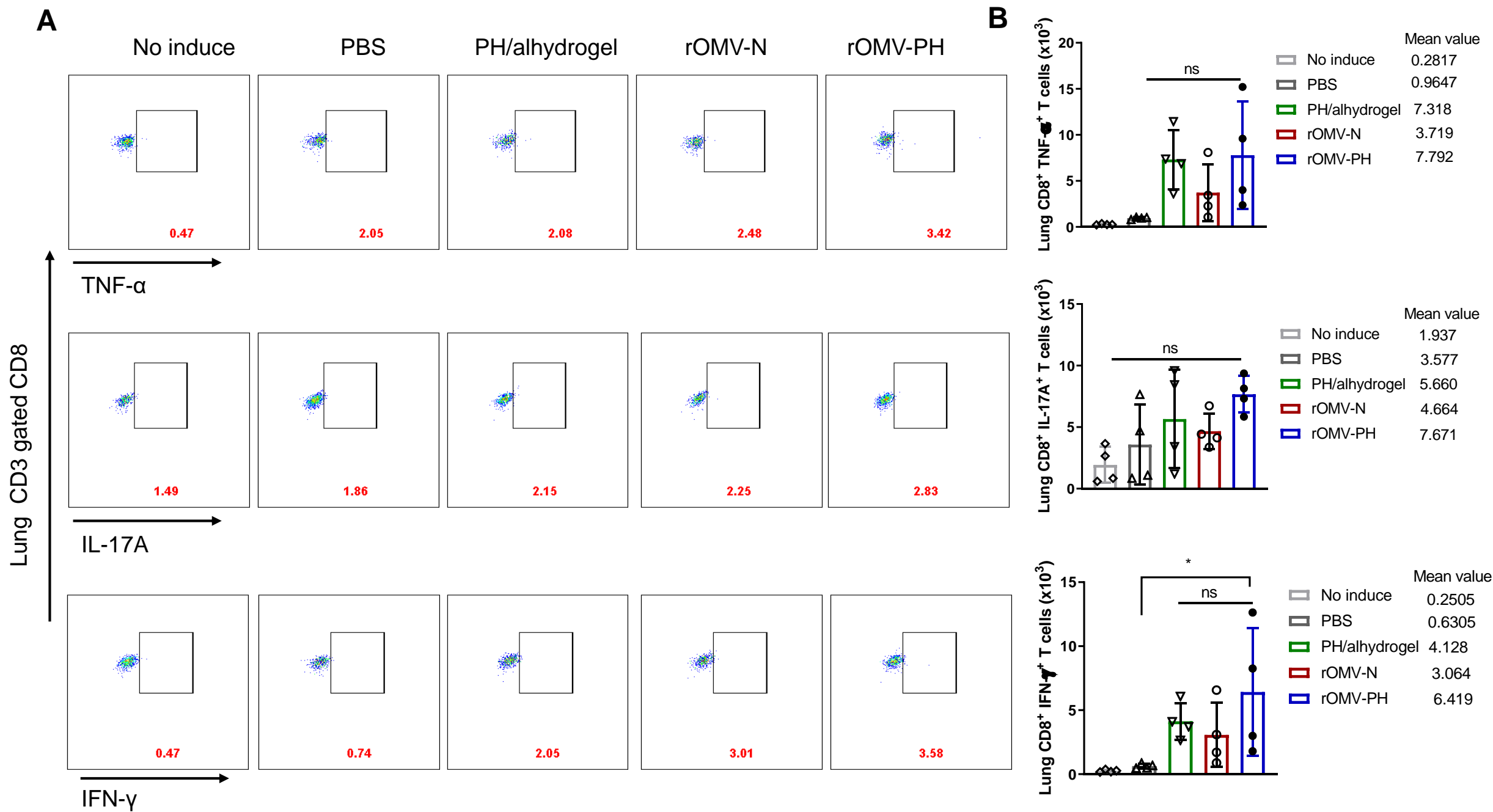

Supplement: FIG S2 [file msphere.00699-21-sf002.pdf]

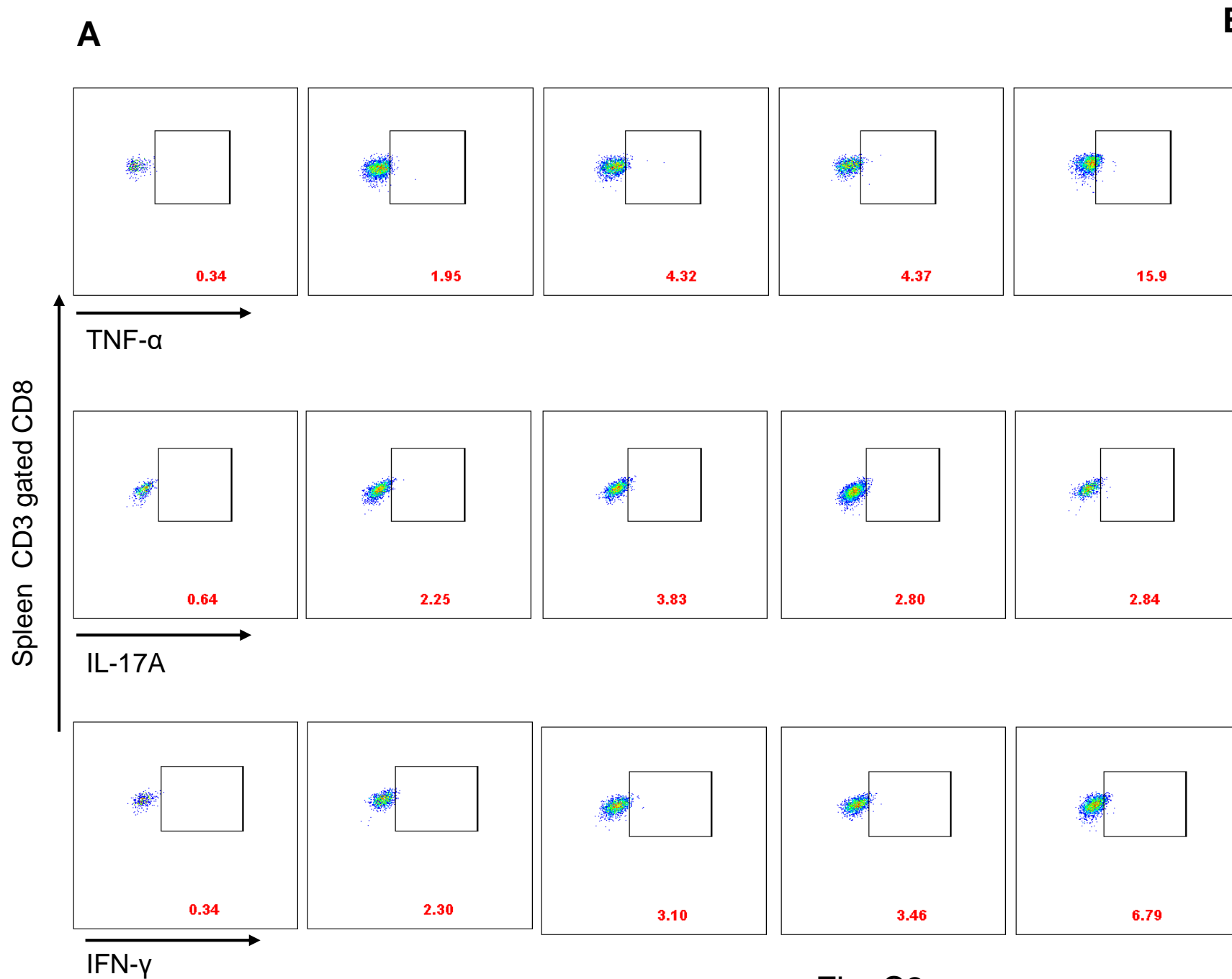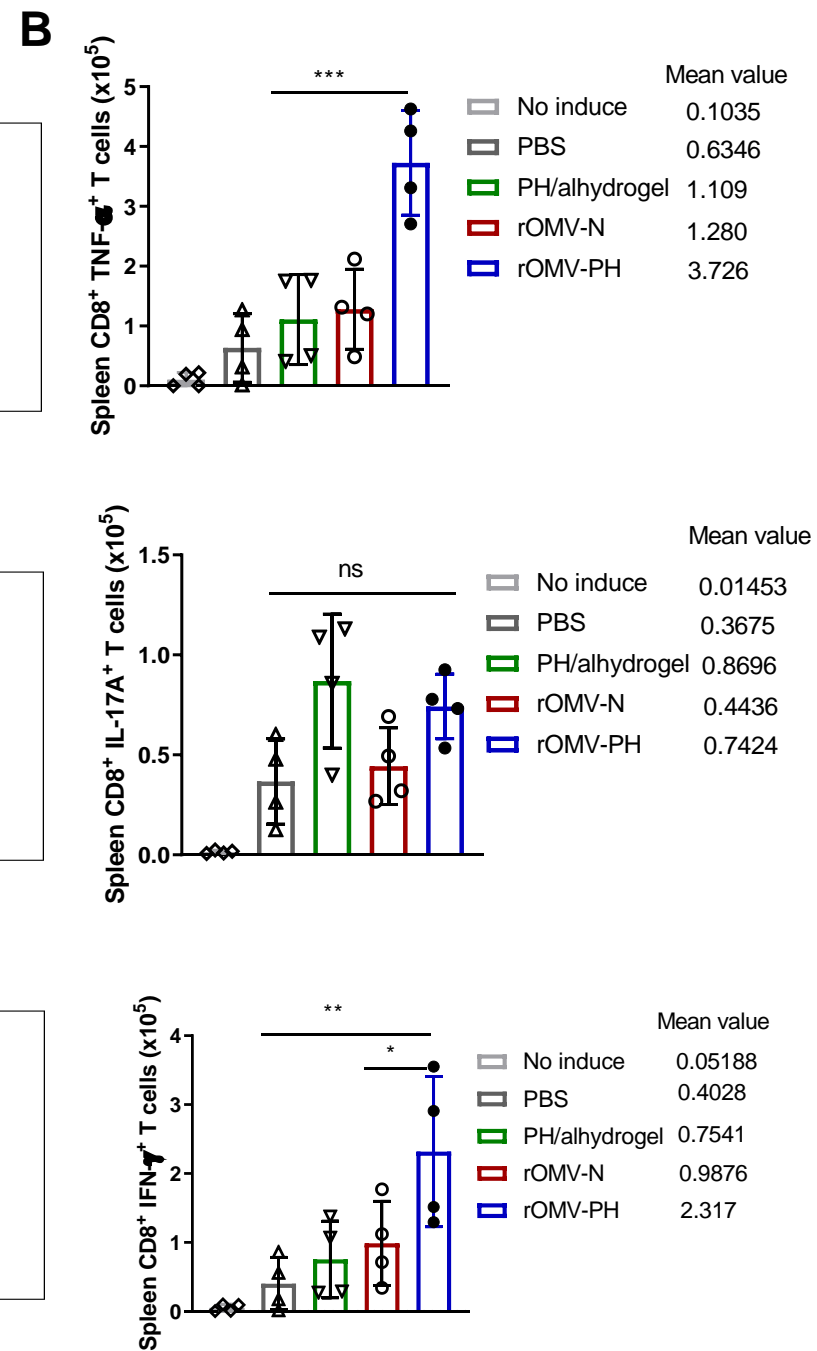

Fig. S3

Supplement: FIG S3 [file msphere.00699-21-sf003.pdf]

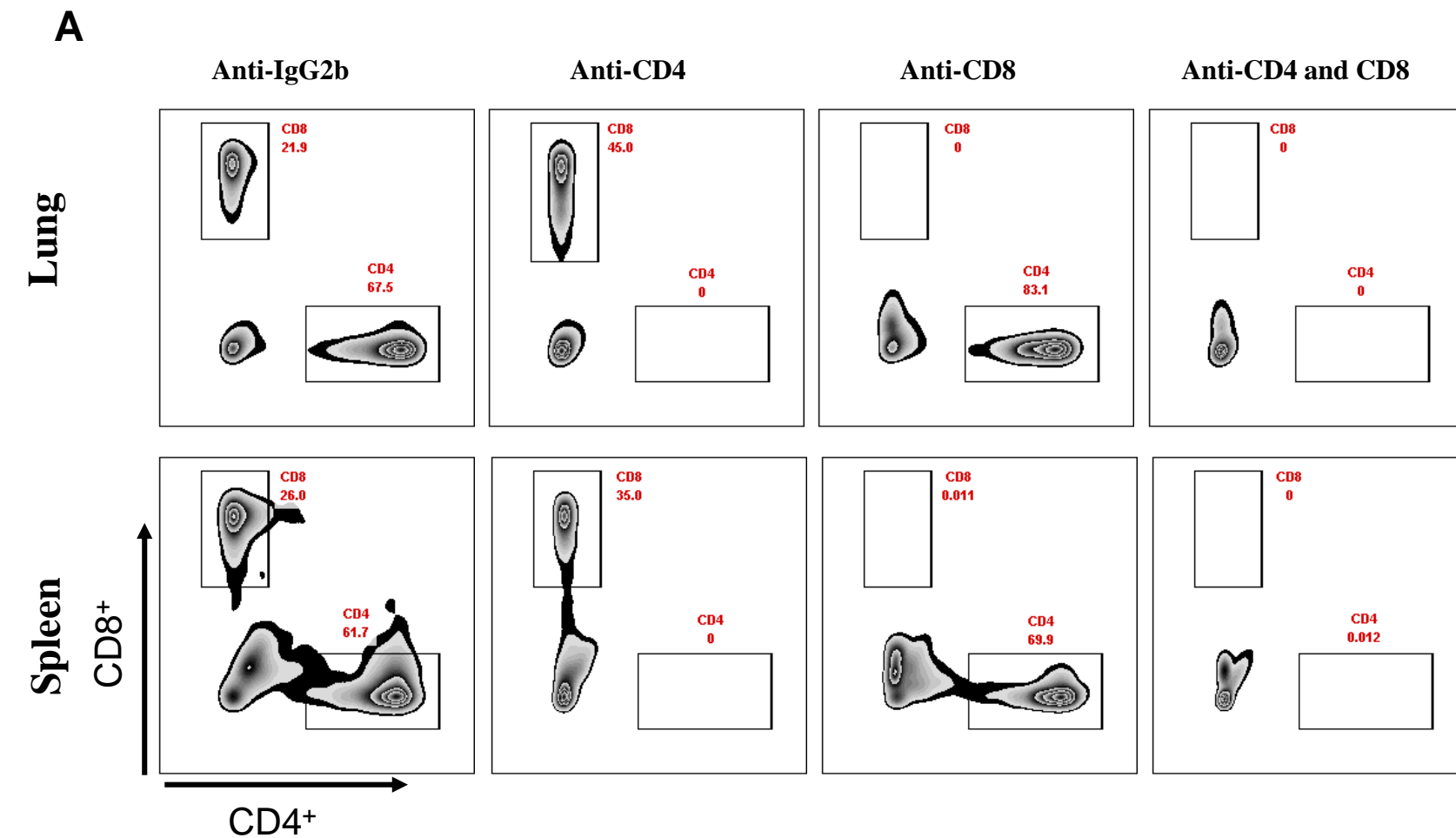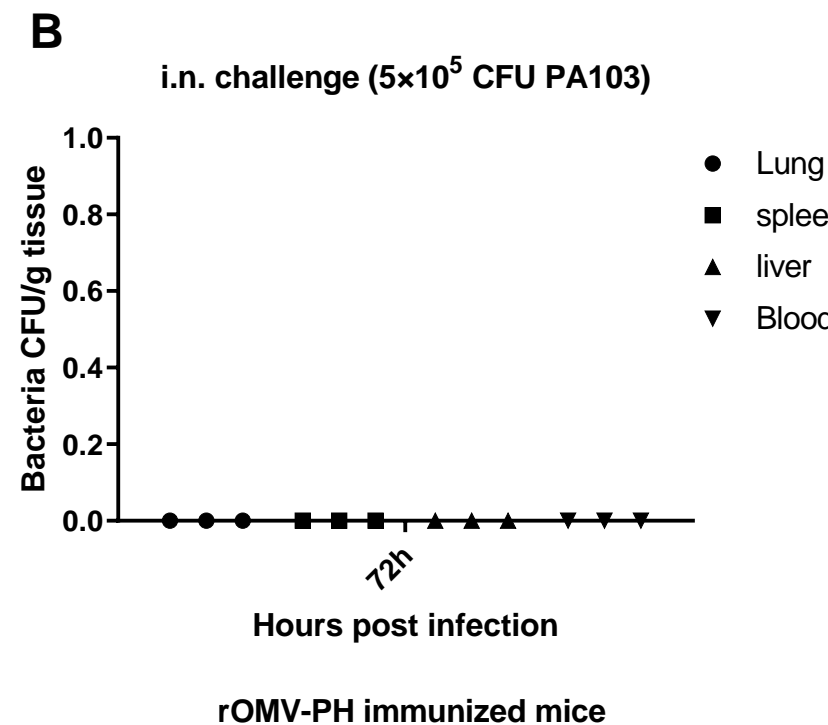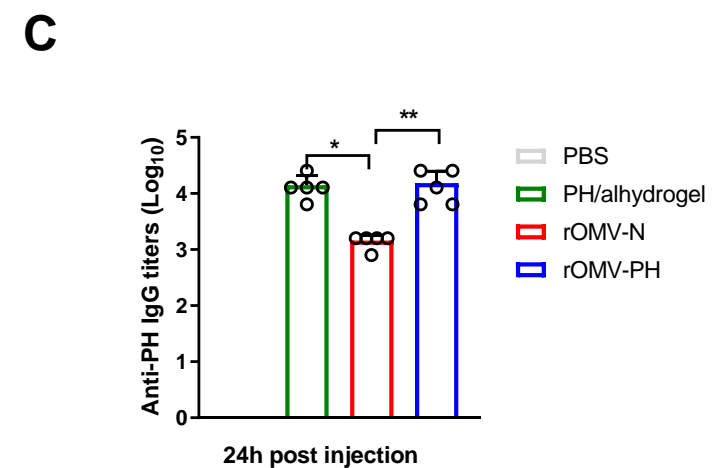

Fig. S4

Supplement: FIG S4 [file msphere.00699-21-sf004.pdf]
