# Supplementary material for: Outer Membrane Vesicles Displaying a Heterologous PcrV-HitA Fusion Antigen Promote Protection against Pulmonary Pseudomonas aeruginosa Infection
Source: mSphere. 2021 Oct 6;6(5):e00699-21. doi: 10.1128/mSphere.00699-21 (PMC8510544; doi:10.1128/mSphere.00699-21)
Supplement: TABLE S1 [file msphere.00699-21-st001.doc]

**Supplementary information (SI)**

**Table S1. Primers used in this work**

| Name | Sequence |
| --- | --- |
| PcrV | cggccatggttgaacaggaagaactgctg (*Nco*I) |
| HitA-His | Cggaagcttttaagtgatgatgatgatgatgtggtgatgatgatg (*Hin*dIII) |
