# Supplementary material for: Outer Membrane Vesicles Displaying a Heterologous PcrV-HitA Fusion Antigen Promote Protection against Pulmonary Pseudomonas aeruginosa Infection
Source: mSphere. 2021 Oct 6;6(5):e00699-21. doi: 10.1128/mSphere.00699-21 (PMC8510544; doi:10.1128/mSphere.00699-21)
Supplement: TEXT S1 [file msphere.00699-21-t0001.doc]

**Supplementary information (SI)**

**MATERIALS AND METHODS**

**Bacterial culture conditions**

All E. coli strains were grown routinely at 37°C in LB broth (1) or LB Agar (Difco). E. coli strain, χ7213 (2) was used to construct suicide vectors and conjugate with Y. pseudotuberculosis (Yptb) for generating mutations. The YptbS44 mutant strain was constructed using Yptb PB1+ derivatives curing the 70 kb pYV plasmid that was described previously (3). Diaminopimelic acid (DAP) at 50 μg/ml, ampicillin at 100 µg/ml, or chloramphenicol at 25 µg/ml was supplemented to media, when necessary.

Fully virulent strains P. aeruginosa PA103 and PAO1 were used for the animal challenge (4). A single colony of PA103 was inoculated in LB broth and grown overnight at 37oC. Bacteria were diluted into 10 ml of fresh LB to obtain a 600 nm of optical density (OD600) of 0.1 and grown at 37oC to an OD600 of 0.6. The cells were then harvested, and the bacterial pellet was resuspended in 1 ml of isotonic PBS and then adjusted to an appropriate concentration for the challenge.

**Molecular procedures**

Plasmids were listed in Table 1 and primers in Table S1. The codon-optimized and truncated pcrV (E28-I294, removing signal peptide) fused with hitA (D28-N355) from PA103 strain together (designated as pcrV-hitAT) for favoring antigen expression in the recombinant Yptb strain was cloned into EcoRI and HindIII sites of pYA3494 to generate the pSMV81 plasmid, in which the pcrV-hitAT was fused with bla ss (encoding β-lactamase N-terminal signal sequence) driven by the Ptrc promoter. The pcrV-hitAT-6xHis gene fragment was amplified from pSMV81 using a PcrV/HitA-his primer set and cloned into NcoI and HindIII sites of pYA3342 to generate pSMV82 plasmid for synthesizing PcrV-HitAT (PH)-His antigen. All the plasmids were confirmed by PCR screening and DNA sequencing.

An asd mutation was introduced into a Yptb mutant strain as described in our previous study to generate the YptbS44 strain (Table 1) which can adapt the Asd+ plasmid pSMV81 (Fig. 1A and Table 1) for over synthesis of the heterologous PH fusion antigen. The procedures for sacB-based sucrose counter-selectable suicide vectors used to construct unmarked deletion and/or insertion mutations in Yptb were described in our previous report (5). Successful gene mutations were confirmed by PCR screening.

**Bacterial subcellular fractionation analysis**

The Yptb mutant strain YptbS44 harboring pSMV13(Bla-V) was grown in HIB at 28°C for 14 h and then incubated at 37°C for 4 h. Bacterial cells were collected by centrifugation (10,000 × g) for 10 minutes. Periplasmic and cytoplasmic fractions were prepared by a lysozyme-osmotic shock method (6, 7). Equal volumes of periplasmic, cytoplasmic, supernatant fractions, and total lysate samples were separated by SDS-PAGE and analyzed for the presence of LcrV or F1 using western blotting.

**OMV isolation**

OMVs were isolated from YptbS44 harboring pYA3493 or pSMV81 following our previous description (8). Briefly, strains were grown at 28°C in LB broth for 16 h. The bacterial cultures were supplemented with EDTA (pH 8.0) at 100 mM and kept on ice for 1 h. Then, the bacterial cells were pelleted by centrifugation at 10,000 × g at 4°C for 20 min. The culture supernatant was filtered using a 0.45 μm pore membrane (Millipore) to remove the residual bacteria and cell debris and then concentrated with a 100 kDa filter using a Vivaflow 200 system (Sartorius). The OMVs were harvested by ultracentrifugation (120,000 × g) for 2 h at 4°C. The vesicle pellet was washed and resuspended in 0.1x sterilized PBS (pH 7.4), and the ultracentrifugation step was repeated. The final vesicle pellet was resuspended in 0.1x sterilized PBS and filtered with a 0.22 μm pore membrane (Millipore) for subsequent experiments.

**Lipid A isolation and analysis by mass spectrometry**

Isolation of lipid A species from Yptb and its OMVs were performed using previously described procedures (9) with minor modifications. Briefly, 5-ml cultures of Yptb were grown at 28°C in LB broth with 180 rpm shaking until an OD600 of ~1.5 was reached. Bacteria were harvested by centrifugation and washed with Milli-Q H2O twice. Bacterial pellets were then resuspended in a single-phase Bligh-Dyer mixture: 5 ml of chloroform, 10 ml of methanol, and 4 ml of H2O (1:2:0.8 v/v/v). The resuspended mixture was vortexed vigorously, incubated at room temperature for 30 min to ensure cell lysis, and centrifuged at 2,000 × g for 20 min. The pellet containing LPS and cell debris was collected and washed once with a single-phase Bligh-Dyer mixture. Then, the pellet was resuspended in 2 ml of 25 mM sodium acetate (pH 4.5) and incubated for 30 min in a boiling water bath to remove the polysaccharide chain from LPS. The hydrolyzed sample was cooled to room temperature and converted into a two-phase Bligh-Dyer mixture by adding 2 ml of chloroform and 2 ml of methanol (chloroform: methanol: H2O, ~2:2:1.8). The sample was mixed by vigorous vertexing and centrifuged for 10 minutes at 2,000 × g. The lower phase (chloroform portion) containing lipid A was transferred into a clean Teflon centrifuge tube using a glass pipet and dried under a stream of nitrogen using a nitrogen dryer. The dried sample was stored at -20°C for mass spectrometry (MS) analysis. For lipid A isolation from OMVs, 2 ml of OMVs isolated from bacteria were initially mixed with 6 ml of chloroform:methanol (1:2, v/v). The same procedures were used for lipid A extraction.

For MS analysis, lipid A extract was resuspended in 100 μl of chloroform-methanol (2:1, v/v), of which 10 μl was injected for each LC/MS analysis. NPLC-ESI/MS was performed as previously described (10, 11) using an Agilent 1200 Quaternary LC system (Santa Clara, CA) coupled to a high-resolution TripleTOF5600 mass spectrometer (Sciex, Framingham, MA). An Ascentis® Si HPLC column (5 μm, 25 cm × 2.1 mm, Sigma-Aldrich) was used. Mobile phase A consisted of chloroform/methanol/aqueous ammonium hydroxide (800:195:5, v/v/v). Mobile phase B consisted of chloroform/methanol/water/aqueous ammonium hydroxide (600:340:50:5, v/v/v/v.). Mobile phase C consisted of chloroform/methanol/water/aqueous ammonium hydroxide (450:450:95:5, v/v/v/v). The elution program was as follows: 100% mobile phase A was held isocratically for 2 min and then linearly increased to 100% mobile phase B for 14 min and held at 100% B for 11 min. The LC gradient was then changed to 100% mobile phase C for 3 min, held at 100% C for 3 min, and finally returned to 100% A over 0.5 min and held at 100% A for 5 min. The instrument settings for negative ion ESI and MS/MS analysis of lipid species were as follows: ion spray voltage (IS) = -4500 V; current gas (CUR) = 20 psi (pressure); gas-1 (GS1) = 20 psi; declustering potential (DP) = -55 V; and focusing potential (FP) = -150 V. The MS/MS analysis used nitrogen as the collision gas. Data acquisition and analysis were performed using Analyst TF1.5 software (Sciex, Framingham, MA).

**Antibody analysis**

The recombinant PH-His protein was purified using the nickel column as described previously for antibody analysis (10). The rPH-specific antibody titers in sera were determined by the enzyme-linked immunosorbent assay (ELISA). The 96-well rPH-coated plates were incubated with two-fold serially diluted serum samples. The colorimetric reaction was developed by alkaline phosphatase-conjugated secondary antibodies in the presence of nitrophenyl phosphate disodium substrate (Sigma). Sera with the highest dilution giving OD405 nm value a minimum of 2-fold higher than sera from PBS-immunized mice were considered as the positive antibody titer.

Stimulation assay in cell lines. To determine the stimulatory activity of OMVs via the Toll-like receptor 4 (TLR4), HEK-BlueTM hTLR4 (InvivoGen, CA, USA) was maintained at 37 °C with 5% CO2 in DMEM (Gibco BRL, Grand Island, NY, USA) containing 10% FBS supplemented with 100 μg/ml penicillin, 100 μg/ml streptomycin, and 100 µg/ml Normocin. Cells were seeded at a density of 5 × 10^4^ cells per well in 96-well tissue culture plates (Costar, Washington, DC) and were stimulated with 20μl OMVs isolated from different strains (final concentration 10 μg/ml) for 8 h. Purified protein and PBS were used as negative controls. Relative NF-κB activity was determined by measuring the embryonic alkaline phosphatase (SEAP) activity in the culture supernatant according to the manufacturer’s instructions.

**Reference**

1. Bertani G. 1951. Studies on lysogenesis. I. The mode of phage liberation by lysogenic Escherichia coli. J Bacteriol 62:293-300.

2. Roland K, Curtiss RI, Sizemore D. 1999. Construction and evaluation of a delta cya delta crp Salmonella typhimurium strain expressing avian pathogenic Escherichia coli O78 LPS as a vaccine to prevent airsacculitis in chickens. Avian diseases 43:429-41.

3. Singh AK, Curtiss R, 3rd, Sun W. 2019. A Recombinant Attenuated Yersinia pseudotuberculosis Vaccine Delivering a Y. pestis YopENt138-LcrV Fusion Elicits Broad Protection against Plague and Yersiniosis in Mice. Infect Immun 87.

4. DiGiandomenico A, Rao J, Harcher K, Zaidi TS, Gardner J, Neely AN, Pier GB, Goldberg JB. 2007. Intranasal immunization with heterologously expressed polysaccharide protects against multiple Pseudomonas aeruginosa infections. Proc Natl Acad Sci U S A 104:4624-9.

5. Sun W, Sanapala S, Henderson JC, Sam S, Olinzock J, Trent MS, Curtiss R, 3rd. 2014. LcrV delivered via type III secretion system of live attenuated Yersinia pseudotuberculosis enhances immunogenicity against pneumonic plague. Infection and immunity 82:4390-404.

6. Witholt B, Boekhout M, Brock M, Kingma J, Heerikhuizen HV, Leij LD. 1976. An efficient and reproducible procedure for the formation of spheroplasts from variously grown Escherichia coli. Anal Biochem 74:160-70.

7. Kang HY, Srinivasan J, Curtiss R, 3rd. 2002. Immune responses to recombinant pneumococcal PspA antigen delivered by live attenuated Salmonella enterica serovar Typhimurium vaccine. Infect Immun 70:1739-49.

8. Wang X, Singh AK, Zhang X, Sun W. 2020. Induction of protective anti-plague immune responses by self-adjuvanting bionanoparticles derived from engineered Yersinia pestis. Infect Immun 88:e00081-20.

9. Hankins JV, Madsen JA, Needham BD, Brodbelt JS, Trent MS. 2013. The outer membrane of Gram-negative bacteria: lipid A isolation and characterization. Methods Mol Biol 966:239-258.

10. Sun W, Curtiss R, 3rd. 2012. Amino acid substitutions in LcrV at putative sites of interaction with Toll-like receptor 2 do not affect the virulence of Yersinia pestis. Microb Pathog 53:198-206.
